# Modulating the catalytic activity of gold nanoparticles using amine-terminated ligands†

**DOI:** 10.1039/d1sc05933e

**Published:** 2021-12-28

**Authors:** Jiangjiang Zhang, Zhentao Huang, Yangzhouyun Xie, Xingyu Jiang

**Affiliations:** Shenzhen Key Laboratory of Smart Healthcare Engineering, Department of Biomedical Engineering, Southern University of Science and Technology No. 1088 Xueyuan Rd., Nanshan District Shenzhen Guangdong 518055 P. R. China jiang@sustech.edu.cn

## Abstract

Nanozymes have broad applications in theranostics and point-of-care tests. To enhance the catalytic activity of nanozymes, the conventional strategy is doping metals to form highly active nanoalloys. However, high-quality and stable nanoalloys are hard to synthesize. Ligand modification is a powerful strategy to achieve chemoselectivity or bioactivity by changing the surface chemistry. Here, we explore different ligands to enhance the catalytic activity of nanozymes, *e.g.*, gold nanoparticles (AuNPs). We systematically studied the impacts on the enzymatic activity of AuNPs by ligand engineering of surface chemistry (charge, group, and surface distance). Our work established critical guidelines for surface modification of nanozymes. The amine group favors higher activity of AuNPs than other groups. The flexible amine-rich ligand enhances the catalytic activity of AuNPs in contrast to other ligands and unmodified AuNPs. Using a proof-of-concept model, we screened many candidate ligands to obtain polyamine-AuNPs, which have strongly enhanced peroxidase-like activity and 100 times enhanced sensitivity compared to unmodified AuNPs. The strategy of enhancing the catalytic activity of AuNPs using ligands will facilitate the catalysis-related applications of nanozymes in biology and diagnostics.

## Abstract 2

Surface ligand engineering can precisely modulate the catalytic activity of nanozymes from inactive to highly active.

## Graphical abstract

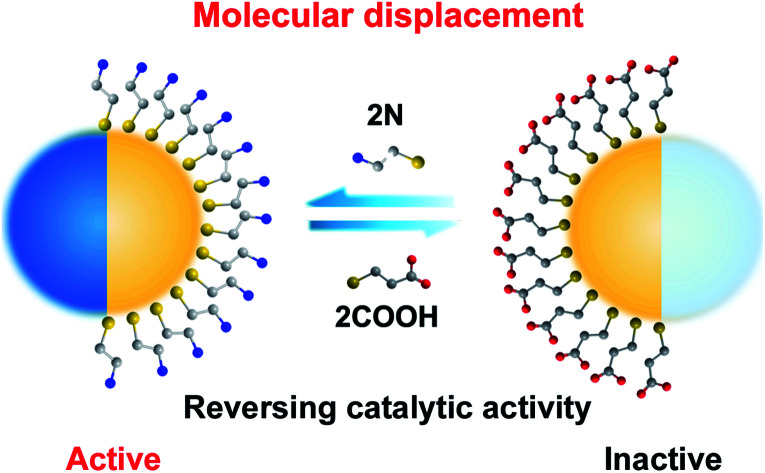

## Introduction

Gold nanoparticles (AuNPs), one of the most explored artificial enzymes, have multiple enzyme-mimic activities, such as peroxidase (POX), glucose oxidase (GOx), and catalase (Cat).^1,2^ The activities make AuNPs powerful candidates for biological and analytical applications.^3–11^ AuNPs have become smart building blocks for the point-of-care test (POCT) and clinical diagnosis. For example, AuNPs catalyze H_2_O_2_ to O_2_ to move the ink bar in the microfluidic chip for the visual POCT.^12^ Owing to their excellent biocompatibility, AuNPs have good renal clearance when the size is <3 nm (called gold nanoclusters, AuNCs).^13,14^ Combining renal clearance and enzymatic activity, AuNCs-tetramer has been designed for *in vivo* diagnosis.^15^ Protease-triggered decomposition generates mono-AuNCs that can pass through the kidney as part of urine for the colorimetric readout of POX-mimic activity.

Surface modification of AuNPs (covalent anchoring or physicochemical adsorption) produces a molecular shell or protein corona to achieve chemoselectivity or specific bioactivity.^3,4,16–20^ Surface ligand-mediated chemistry plays an important role in their catalytic activity.^21–23^ Few studies have described phenomena such as self-catalysis and self-limiting activity of AuNPs, light-triggered plasmonic excitation of catalysis, electrostatic neutralization-controlled catalysis, molecular imprinting-induced selective catalysis, and metal ion-assisted catalysis.^24–30^ Our previous study demonstrated the self-catalyzed *in situ* deposition of AgNPs on the surfaces of polydopamine-coated AuNPs.^31^ The corona-like AgNPs@AuNPs show a spectral blue-shift and high activity. In subsequent work, we described the surface-blocking effect of dense packing molecules (cetyltrimethylammonium bromide, CTAB) on the surfaces of AuNPs.^32^ Ag^+^ can break the CTAB-gate to release the POX-mimic activity of AuNPs. However, there was no systematic study to understand how different factors of ligands can affect surface chemistry and thus the catalytic activity of AuNPs. Enhancing the catalytic activity of nanozymes is a momentous and challenging task. The conventional strategy is doping metals to form highly active nanoalloys.^33–37^ However it is hard to scale up the synthesis of high-quality and stable nanoalloys. Exploring a new strategy to enhance the catalytic activity of nanozymes is urgent and promising.

**Fig. 1:**
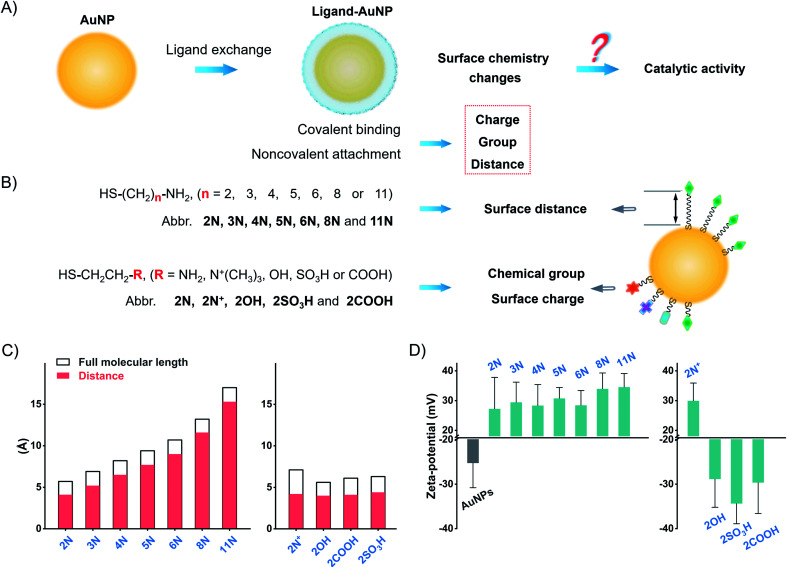
Proposed implications of surface chemical manipulation of AuNPs. (A) and (B) Schematic illustration of different ligand-coated AuNPs and the ligand-simulated surface chemistry of the surface distance, surface group, and surface charge to study the implications on the enzyme-mimic activity of AuNPs. (C) Calculated molecular full length of different ligands and the inter-distance between thiol and terminal groups. (D) Zeta-potential values of AuNPs and different ligands-AuNPs.

Herein, we explored the straightforward pathway to enhance the catalytic activity of nanozymes by surface ligand engineering. We synthesized different alkanethiol ligand-modified AuNPs to investigate the implications of surface chemistry on the catalytic activity of AuNPs. These ligands, with different alkyl chains and terminal groups, have a precise adjustment of surface chemistry on the surface charge, group, and surface distance (Fig. 1). We deciphered the complex interrelations of different factors to accurately modulate the catalytic activity of AuNPs. The surface chemistry studies suggest that the flexibly attached amine ligand favors high catalytic activity of AuNPs compared to the rigidly anchored amine ligand and other ligands. Following this pattern, we compared monoamine, binary amine, and polyamine ligands to find that the increase of amine groups in the ligand results in higher activity of AuNPs. The flexibly attached polyamine ligands significantly enhance the catalytic activity of AuNPs in contrast to unmodified AuNPs, which are normally treated as the most active AuNPs. Testing the POX-mimic activity, the polyamine-AuNPs show 100-times enhanced sensitivity to hydrogen peroxide compared to the unmodified AuNPs. By combining the GOx-linked cascade reaction, the polyamine-AuNPs can quantitate glucose at the micromolar level.

## Results and discussion

We synthesized AuNPs by using ascorbic acid as the reducing and stabilizing agent. AuNPs exhibit a narrow size distribution of 21.8 ± 2.1 nm (PDI = 0.156, Fig. S1–S4†). Since Au^3+^/Au^+^ catalyzes the oxidation by Fenton-like reaction,^38,39^ we use a centrifuge to remove residual gold ions. The centrifuged AuNPs show a weaker POX-mimic activity than the raw product AuNPs (Fig. S5†). AuNPs are used instead of normal citrate stabilized-AuNPs to avoid aggregation when preparing ligand-modified AuNPs (Fig. S6 and S7†).

Ligand exchange produces thiol-modified AuNPs by forming a stable metal–sulfur bond.^40–42^ We selected alkanethiols to precisely manipulate the surface chemistry. Amine alkanethiols with different alkyl carbon numbers (2–11, 2N, 3N, 4N, 5N, 6N, 8N, and 11N) on the surfaces of AuNPs allow angstrom scale adjustment of the surface distance (distance between the active gold surface and ligand tail, which we call “surface distance”, 0.4–1.6 nm, Fig. 1C and Table S2†). Heterobifunctional ethanethiol with primary amine (2N), quaternary ammonium (2N^+^), hydroxyl (2OH), carboxyl (2COOH), and sulfonic (2SO_3_H) groups allows the adjustment of chemical groups and thus surface charges when ionized in water (Fig. 1C and Table S3†). By simply mixing the ligands with AuNPs and incubating them overnight, we obtained different ligand-coated AuNPs.

Ligand modification generates a spectral red-shift and absorbance change of AuNPs. As the alkyl chain length increases, the absorption peak of amine-AuNPs shifts to a longer wavelength (Fig. S8†). For ethanethiols with a fixed alkyl chain, the change in chemical groups and surface charges produces distinct spectral shifts (Fig. S9†). Amine ligands and 2N^+^ reverse the surface charge of AuNPs from negative to positive (Fig. 1D and S10†). 2COOH, 2OH, and 2SO_3_H increase the negative zeta-potential of AuNPs. These zeta-potential changes further prove the modification of different ligands on the surfaces of AuNPs. Successful modification of these ligands also changes the conductivity of the AuNP solution (Fig. S11†). X-ray photoelectron spectroscopy (XPS) profiles of different ligands-AuNPs show binding energy peaks: S2p ∼ 162 eV (Au–S bond), S2p ∼ 168 eV (–SO_3_H), N1s ∼ 400 eV (–NH_2_), and N1s ∼ 402 eV (–N^+^(R)_3_) (Fig. S12†). These results indicate that those ligands anchored on the surfaces of AuNPs.

To study the surface coverage of AuNPs, we selected 11N as a characterization model. 11N can form a self-assembly monolayer (SAM) on the metal surface.^43–45^11N on the surfaces of AuNPs produces a spectral shift and absorbance change (Fig. S13†). XPS elemental analysis can quantify the relative amount of ligands and assess the surface capping efficiency.^46,47^ However, the tests of nanomaterials are not stable enough and highly dependent on skillful operations, due to the trace amount of ligands (especially when the relative element amount is <5%). We thus use a straightforward *in situ* characterization method to provide the authentic evaluation of surface coverage.^48^

**Fig. 2:**
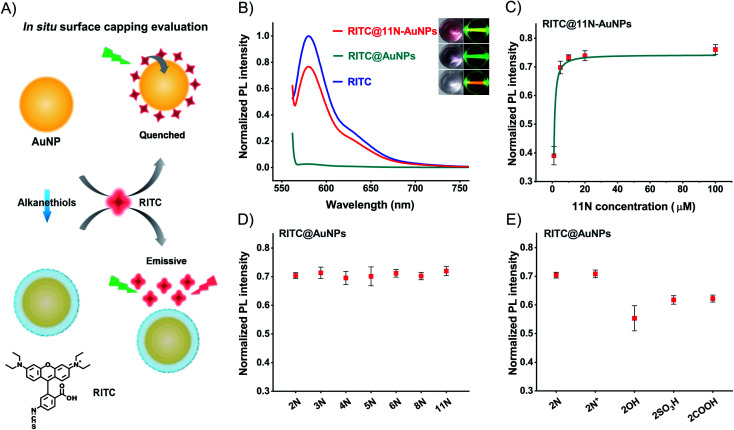
Characterization of the surface coverage of AuNPs. (A) Schematic illustration of AuNP-caused fluorescence quenching of RITC and thiol ligand-mediated prevention of quenching. (B) Fluorescence spectra of RITC, RITC@AuNPs, and RITC@11N-AuNPs. Inset: photographs of different solutions under daylight and green-laser (532 nm) irradiation (RITC, 2 μM). (C) Normalized fluorescence intensity of RITC@different concentrations of 11N modified AuNPs. (D) Normalized fluorescence intensity of RITC@AuNPs and RITC@different amine-AuNPs. (E) Normalized fluorescence intensity of RITC@different ethanethiol-AuNPs.

We use the fluorescence change of an *in situ* quenching system to evaluate the surface coverage (Fig. 2A). AuNPs quench fluorophores attached to their surface, owing to the surface plasmon resonance and energy transfer. Fluorescent Rhodamine B isothiocyanate (RITC, Fig. S14†) binds to AuNPs and AuNPs quench its fluorescence.^49^ Thiol molecules like 11N prevent RITC from binding to the surfaces of AuNPs. The RITC@11N-AuNP mixture is emissive. By lighting with a green laser, we could visually tell the difference between AuNPs and 11N-AuNPs in the presence of RITC (Fig. 2B). 11N also prevents AuNPs from RITC-induced aggregation (Fig. S15†). It is only the complete surface coverage that can fully block the interaction between RITC and AuNPs. We utilized the fluorescence of RITC@ligand-AuNPs *via* that of RITC@AuNPs to evaluate the surface coverage. This *in situ* evaluation strategy is reliable and appropriate for studying the surface chemistry of nanomaterials.

The quenching is complete at concentrations of RITC ≤2 μM (Fig. S16†). Owing to the extinction of AuNPs, the overlap between the absorption of AuNPs and the emission of RITC causes a lower fluorescence of the RITC@11N-AuNP mixture than the RITC by itself (Fig. 2B). 100 μM 11N, about 10^6^-times higher than AuNPs, is treated to cover the surface of AuNPs completely. The POX-mimic activity of 11N-AuNPs is fully inhibited. After testing different amounts of 11N, it was found that 10 μM satisfies the complete surface coverage of AuNPs (Fig. 2C and S17†). The calculated ligand concentration to achieve the complete surface coverage is about 6.35 μM (ESI,† calculation of the surface coverage of AuNPs). 10 μM thiol ligands are used to ensure maximum surface coverage. At this concentration, all thiol ligands with different alkyl chain lengths (from 2 to 11) achieve complete surface coverage (calculated ligand density about 15 nm^−2^) to block the quenching of RITC (Fig. 2D). Once the blocking effect is established, it has no significant change with respect to the alkyl chain lengths, since the interaction between RITC and AuNPs is completely blocked by bonded ligands. For ethanethiol ligands, 2COOH and 2SO_3_H produce a slightly lower fluorescence owing to the electrostatic binding between 2COOH/2SO_3_H-AuNPs and RITC (Fig. 2E). 2OH-AuNPs are the aggregate having high absorption at 580 nm (Fig. S9†), to result in a lower fluorescence. Owing to the same blocking effect and the spectral overlap between AuNPs and RITC, 2N^+^ and 2N have similar fluorescence recovery. The above surface ligand studies demonstrated a complete surface modification of AuNPs.

**Fig. 3:**
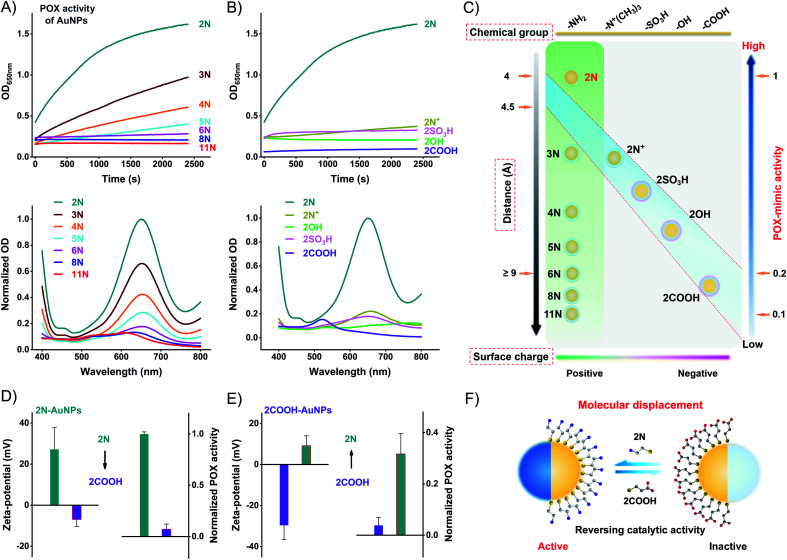
Ligand-mediated surface chemistry impacts on the POX-mimic activity of AuNPs. (A) Top: distance effects by kinetic monitoring of the absorption of different amine-AuNPs upon incubation with TMB/H_2_O_2_. Bottom: the corresponding endpoint spectra. (B) Top: chemical group and surface charge effects by kinetic monitoring of the absorption of different ethanethiols-AuNPs upon incubation with TMB/H_2_O_2_. Bottom: the corresponding endpoint spectra. (C) Graphical illustration of the interactional relationship of ligand-simulated surface chemistry on impacting the POX-mimic activity of AuNPs. (D) Zeta-potential change and POX-mimic activity change of 2N-AuNPs after incubation with 2COOH (200 μM). (E) Zeta-potential change and POX-mimic activity change of 2COOH-AuNPs after incubation with 2N (200 μM). (F) Schematic illustration of the molecular effects of the catalytic activity of AuNPs by ligand displacement.

Using the commercial 3,3′,5,5′-tetramethylbenzidine (TMB)/H_2_O_2_ substrate, we compared the POX-mimic activity of different ligand-coated AuNPs. Amine ligands generate a positive surface charge of AuNPs. They provide a single variation of surface distance which corresponds to the alkyl chain length. For 2N to 11N, the distance ranges from 0.41 to 1.53 nm (Table S2†). The distance isolates the active Au surface from substrates,^32^ thus hindering the catalytic activity of AuNPs. A longer distance results in lower POX-mimic activity. 2N makes the smallest distance of 0.41 nm, showing the highest activity. 6N-AuNPs have a distance of ∼0.9 nm while losing over 90% activity (Fig. 3A). Ethanethiol ligands have a similar distance of 0.41–0.44 nm (Table S3†). They are treated as a fixed distance to provide changes of the surface group and surface charge. Positively charged 2N/2N^+^-AuNPs have higher POX-mimic activity than negatively charged 2OH/2SO_3_H/2COOH-AuNPs (Fig. 3B). 2COOH-AuNPs are inactive with extremely weak activity. The positive 2N^+^-AuNPs have much lower activity than 2N-AuNPs. They have the same positive charge but amine has a smaller steric hindrance. Besides the surface charge, the different surface groups strongly regulate the activity of AuNPs as well. At the fixed surface distance, the surface charge and group changes dramatically impact the catalytic activity of AuNPs. 2N-AuNPs have the highest activity, which shows that the amine group favors high catalytic activity.

The catalytic activity of AuNPs is highly dependent on their surface chemistry (Fig. 3C). AuNPs can be modulated between active and inactive states by ligand displacement. We incubated 2N-AuNPs with excess 2COOH. 2COOH displacement produces an opposite surface charge (Fig. S18†). It strongly reverses 2N-AuNPs from being active to inactive. 2COOH-displaced 2N-AuNPs lose POX-mimic activity (Fig. 3D). To test whether the inactive AuNPs can be converted to active AuNPs by ligand displacement, we incubated 2COOH-AuNPs with excess 2N. The opposite surface charge indicates successful ligand displacement (Fig. S18†). 2N-displaced 2COOH-AuNPs show significant POX-mimic activity in contrast to the inactive 2COOH-AuNPs (Fig. 3E). Molecular conversion between 2N and 2COOH reversely modulated the catalytic activity of AuNPs. AuNPs can be a powerful candidate for stimuli-responsive systems for bioorthogonal chemistry and analytical sensing.

**Fig. 4:**
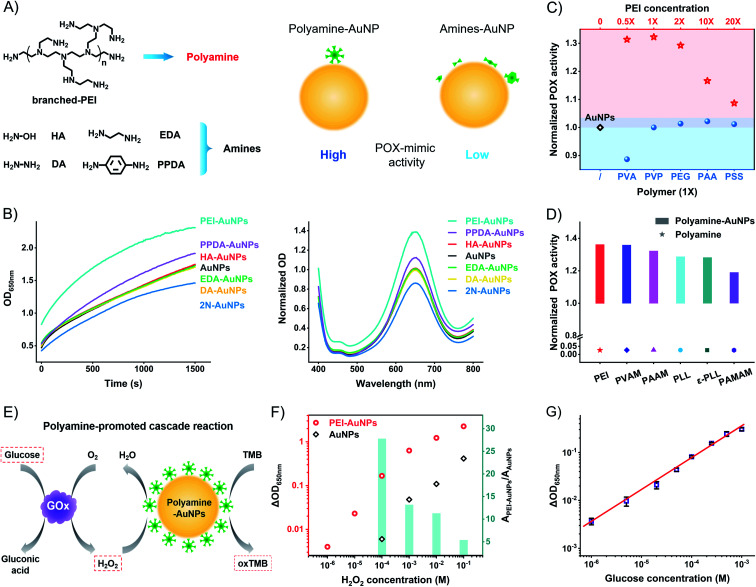
Polyamine ligand to promote the POX-mimic activity of AuNPs. (A) Schematic illustration of the effect of flexibly attached amine ligands on the POX-mimic activity of AuNPs. The red arrow means an enhancement of the catalytic activity of polyamine-AuNPs compared to amine-AuNPs. (B) POX-mimic activity tests of AuNPs and amine-AuNPs by kinetic monitoring and the endpoint spectra. (C) Normalized POX-mimic activity of AuNPs (black diamond), different amounts of PEI-modified AuNPs (red stars), and different polymer-modified AuNPs (blue circles). 1X = 3 μg mL^−1^. (D) Normalized POX-mimic activity of different polyamine-AuNPs and the polyamine itself. (E) Schematic illustration of the polyamine-promoted cascade reaction for the detection of H_2_O_2_ and glucose. (F) Concentration-dependent absorption changes of AuNPs and PEI-AuNPs when responding to H_2_O_2_. Inset of the column chart: the corresponding ratio of absorption changes of AuNPs and PEI-AuNPs responding to the same amount of hydrogen peroxide. (G) Linear fitting curve of optical absorption changes of PEI-AuNPs and GOx (0.5 mg mL^−1^) when responding to different concentrations of glucose. The data indicate three duplications.

The above model studies demonstrate the benefit of the amine group and the harm of the surface distance to the high catalytic activity of AuNPs. The metal–sulfur bond anchored ligands form SAMs to produce a rigid space on the surfaces of AuNPs with the blocking effect. To compromise the conflict between the amine group and rigid surface space, the weakly attached amine ligands to generate flexible space on the surfaces of AuNPs are the strong candidates for high catalytic activity. We tested the weakly attached amine ligands, including hydroxylamine (HA), diamine (DA), ethylenediamine (EDA), *p*-phenylenediamine (PPDA), and branched polyethyleneimine (PEI) (Fig. 4A). These amine ligands have a dynamic interaction of attaching-and-detaching on the surfaces of AuNPs, thus not forming a rigid space with a blocking effect. Compared to unmodified AuNPs, HA, DA, and EDA have no significant influence on the catalytic activity (Fig. 4B). Binary amine ligand PPDA increases the POX-mimic activity of AuNPs. At the same time, they all have higher catalytic activity than 2N-AuNPs. Flexible amine ligands result in higher catalytic activity of AuNPs in contrast to rigid amine ligands.

These results led us to test other amine-rich (polyamine) ligands such as PEI, which strongly enhance the POX-mimic activity of AuNPs. PEI with different molecular weights has similarly enhanced activity (Fig. S19†). The enhancement is concentration-dependent (Fig. 4C and S20†). Moderate amounts of PEI on the surfaces of AuNPs exhibit maximum catalytic activity. Too high or too low concentration of PEI on the surfaces of AuNPs results in compromised activity. We believe that PEI with too high density results in a crowded space, and causes the weakening of POX-mimic activity. Based on the XPS tested surface N : Au ratio and calculations,^50–52^ the surface N element density of PEI-AuNPs is about 78 nm^−2^ (Table S1†). It is 5.3 times higher than that of rigid amine ligands. High-resolution XPS profiles verify the PEI ligand on the surfaces of AuNPs (Fig. S21a†). Comparing the Au4f profiles of AuNPs and PEI-AuNPs, the binding energy peaks of 83.9 eV (Au^0^4f_7/2_) and 87.6 eV (Au^0^4f_5/2_) have no shift (Fig. S21b†). There is no Au^3+^ or Au^+^ ion release from PEI-AuNPs. To further verify the polyamine-enhanced catalytic activity, we tested polyvinylamine (PVAM), polyallylamine (PAAM), poly-l-lysine (PLL), ε-poly-l-lysine (ε-PLL), and polyamidoamine (PAMAM) dendrimers (Fig. S22†). These amine-rich polymers increase the POX-mimic activity of AuNPs (Fig. 4D and S23†). The enhancement is connected to the N element density on the surface of AuNPs (PEI > PVAM > PAAM > PLL ≈ ε-PLL > PAMAM, Table S1†), while for different functional polymers, including polyvinyl alcohol (PVA), polyethylene glycol (PEG), polyvinylpyrrolidone (PVP), polyacrylic acid (PAA), and polystyrene sulfonate (PSS), they barely enhance the catalytic activity of AuNPs (Fig. 4C and S24†). PEI and these polymers themselves show no catalytic activity (Fig. S25 and S26†). To study the size effect of AuNPs, we further used the ∼13 nm AuNPs^53^ and ∼5 nm AuNPs.^32^ A similar enhancement of POX-mimic activity of different size AuNPs is observed in the presence of PEI (Fig. S27†). To study the mechanisms of the enhanced catalytic activity of polyamine-AuNPs, we tested the oxygen radicals involved in the catalysis. Nanozymes like AuNPs catalyze hydrogen peroxide to produce more activated reactive oxygen species (ROS) such as hydroxyl radicals (·OH), which respond to the oxidation of other substrates.^54–57^ PEI-AuNPs generate more ^**·**^OH compared to AuNPs and EDA-AuNPs (Fig. S28†). These results may suggest that the polyamine ligand with increased amine (electron donors) density on the surfaces of AuNPs facilitates the conversion of ROS. This phenomenon is possibly like the doping effect in nanozymes (such as phosphor/nitrogen doping).^58–60^

Polyamine ligands, with a great density of amine groups and flexible space on the surfaces of AuNPs, amplify the catalytic activity of AuNPs. PEI-AuNPs are more sensitive to respond to H_2_O_2_ in contrast to unmodified AuNPs. At the same concentrations, PEI-AuNPs produce a higher signal (absorption change at 650 nm) than unmodified AuNPs (Fig. 4F and S29†). The enhancement is up to 27.8 times at 100 μM H_2_O_2_. The detectable value of H_2_O_2_ is 100 times lower than that of unmodified AuNPs. We further compared 2N-AuNPs and EDA-AuNPs. Responding to the same amount of H_2_O_2_, PEI-AuNPs have the highest signal as well (Fig. S30†). PEI-AuNPs show excellent stability compared to horseradish peroxidase (HRP). 30 mU mL^−1^ HRP shows similar catalytic activity to PEI-AuNPs (Fig. S31†). The temperature-dependent curves of POX activity reveal that the half-maximum inhibition temperature (IT_50_) is 31 °C for HRP and 45 °C for PEI-AuNPs (Fig. S32†). HRP is fully inactivated at 45 °C. PEI-AuNPs have good temperature tolerance and maintain the activity even at 80 °C. The time-dependent curves show the POX activity attenuation of HRP during 2 weeks at room temperature (Fig. S33†). PEI-AuNPs are consistent without apparent POX activity decay. PEI-AuNPs are more compatible than HRP for decentralized applications and underdeveloped conditions. Coupling with the GOx-linked cascade reactions, we optimized the PEI-AuNP system to detect glucose. The linear fitting range covers three orders of magnitude (from 1 μM to 1 mM, Fig. 4G and S34†). The calculated limit of detection (LOD) value is about 0.78 μM (3*σ*/slope). The LOD value and detection range are superior to those of lots of glucose POCT sensors (Table S4†).

## Conclusions

In conclusion, our systematic studies demonstrate that surface ligand-mediated manipulation of surface chemistry can precisely regulate the catalytic activity of AuNPs. Ligand engineering-introduced changes of charges, groups, and surface distance have different implications on catalytic activity. Molecular displacement of ligands reversely modulates AuNPs between active and inactive states. AuNPs may develop into a powerful candidate for stimuli-responsive systems for bioorthogonal chemistry and analytical sensing. Amine-rich ligands greatly enhance the catalytic activity of AuNPs by increasing the amine density on the surfaces of AuNPs. The ligand modification-enhanced catalytic activity of nanozymes makes it a promising strategy to develop excellent artificial enzymes. The ligand@nanozyme with greatly improved stability and catalytic activity will be a strong candidate to replace natural enzymes. Our work provides critical guidelines for the modification of nanozymes, and will facilitate catalysis-related applications in biology and diagnostics.

## Data availability

Data associated with this article, including synthetic details, materials characterization and ligand calculation details are available in the ESI.†

## Author contributions

J. Zhang designed and conducted the experimental studies, and wrote the manuscript. Z. Huang synthesized the molecular ligands. Y. Xie helped with the surface ligand calculation and discussion. X. Jiang has mentored the whole project. All authors discussed the results and contributed to the manuscript.

## Conflicts of interest

There are no conflicts to declare.

## Supplementary Material

SC-013-D1SC05933E-s001
