# Supplementary material for: Modulating the catalytic activity of gold nanoparticles using amine-terminated ligands
Source: Chem Sci. 2021 Dec 28;13(4):1080–7. doi: 10.1039/d1sc05933e (PMC8790798; doi:10.1039/d1sc05933e)
Supplement: SC-013-D1SC05933E-s001 [file SC-013-D1SC05933E-s001.pdf]

Electronic Supplementary Material (ESI) for Chemical Science.
This journal is © The Royal Society of Chemistry 2021

Supplementary Information

for

Modulating the catalytic activity of gold nanoparticles using amine-terminated ligands

Jiangjiang Zhang,[†] Zhentao Huang,[†] Yangzhouyun Xie,[†] and Xingyu Jiang*,[†]

[†] Shenzhen Key Laboratory of Smart Healthcare Engineering, Department of Biomedical Engineering, Southern University of Science and Technology, No. 1088 Xueyuan Rd., Nanshan District, Shenzhen, Guangdong, 518055, P. R. China

*E-mail: jiang@sustech.edu.cn

Materials list and abbreviations:

Material	Abbreviation
ascorbic acid	AA
sodium citrate	Cit
Rhodamine B isothiocyanate	RITC
3,3',5,5'-tetramethylbenzidine	TMB
2-aminoethanethiol	2N
3-aminopropane-1-thiol	3N
4-aminobutane-1-thiol	4N
5-aminopentane-1-thiol	5N
6-aminohexane-1-thiol	6N
8-aminooctane-1-thiol	8N
11-aminoundecane-1-thiol	11N
2-mercapto-N,N,N-trimethylethanaminium	$2N^{+}$
2-mercaptoethanol	2OH
3-mercaptopropanoic acid	2COOH
2-mercaptoethanesulfonic acid	$2SO_3H$
hydroxylamine	HA
diamine	DA
ethanediamine	EDA
phenylenediamine	PPDA
polyvinyl alcohol	PVA
polyvinyl pyrrolidone	PVP
polyethylene glycol	PEG
polyacrylic acid	PAA
sodium polystyrenesulfonate	PSS
polyallylamine	PAAM
branched-polyethyleneimine	PEI
Polyvinylamine	PVAM
poly-L-lysine	PLL
ε-poly-L-lysine	ε-PLL
Glucose oxidase	GOx
horse radish peroxidase	HRP

Experimental section:

Materials and reagents. $HAuCl_4$ was purchased from Sinopharm Chemical Reagent Co., Ltd. (Shanghai, China). 2-mercapto-N, N, N-trimethylethanaminium (**$2N^+$**), 3,3',5,5'-tetramethylbenzidine (TMB), Rhodamine B isothiocyanate (RITC), polyvinyl alcohol (PVA), polyvinyl pyrrolidone (PVP), polyethylene glycol (PEG), polyacrylic acid (PAA), sodium polystyrenesulfonate (PSS), polyallylamine (PAAM), and horse radish peroxidase (HRP) were purchased from Macklin (Shanghai, China). Glucose, trisodium citrate, ascorbic acid (AA), hydroxylamine (HA), diamine (DA), ethanediamine (EDA), p-phenylenediamine (PPDA), 2-mercaptoethanesulfonic acid (**$2SO_3H$**), 2-mercaptoethanol (**2OH**), 3-mercaptopropanoic acid (**2COOH**), poly-L-lysine (PLL), ε-poly-L-lysine (ε-PLL), 30% H_2O_2 solution, and commercial substrate solution (TMB/H_2O_2) were purchased from Aladdin (Shanghai, China). 2-aminoethanethiol (cysteamine, **2N**), 3-aminopropane-1-thiol (**3N**), 6-aminohexane-1-thiol (**6N**), 8-aminooctane-1-thiol (**8N**), and polyamidoamine (PAMAM) dendrimer were purchased from Sigma-Aldrich (St. Louis MO, USA). Branched polyethyleneimine (PEI) with different average molecular weight (600, 1800, 5000, 10000, and 70000) were purchased from Alfa Aesar (Shanghai, China). 11-aminoundecane-1-thiol (**11N**) purchased from Dojindo Laboratories (Kumamoto, Japan). Polyvinylamine (PVAM) was purchased from Polysciences (Warrington PA, USA). Glucose oxidase (GOx) was purchased from Heowns (Tianjin, China).

Characterizations and instruments. Transmission electron microscope (TEM), high-resolution TEM (HRTEM), and fast Fourier transform (FFT) pattern characterizations were performed with a Tecnai G2 F30 field-emission TEM (FEI, USA). UV-visible optical spectra and kinetic monitoring data were recorded with a UV-2600 spectrophotometer (Shimadzu, Japan) or an Epoch microplate spectrophotometer (BioTek Instruments, USA). Excitation and emission spectra were recorded with an RF-6000 fluorescence spectrophotometer (Shimadzu, Japan). Dynamic light scattering (DLS) size profile and zeta-potential distribution profiles were measured with a Zetasizer Nano ZS instrument

(Malvern, UK). X-ray photoelectron spectroscopy (XPS) data were collected with a Thermo Scientific K-Alpha+ instrument (ThermoFisher, USA).

Synthesis of 4-aminobutane-1-thiol (4N) and 5-aminopentane-1-thiol (5N)

The synthesis of **4N** and **5N** was referred to the published procedure.[1]

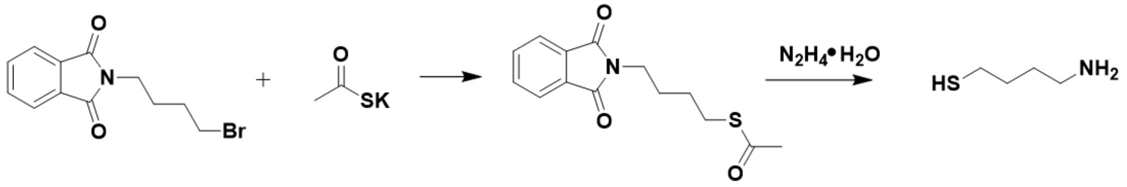

Synthesis of 4-aminobutane-1-thiol: 2-(4-bromobutyl)isoindoline-1,3-dione (2.43 g, 8.6 mmol) was suspended in 40 mL of anhydrous THF (30 mL) and added potassium thioacetate (0.98 g, 8.6 mmol) into the solution. The mixture was refluxed 24 hours. After cooling to room temperature, the mixture was added into H_2O (30 mL), and the aqueous solution was extracted with EtOAc. The combined organic phases were dried with Na_2SO_4. The solvent was removed under reduced pressure to give the solid product (2.38 g, 99%), which was used directly without further purification. Hydrazine hydrate (1.4 mL, 36.4 mmol) was added into a solution of S-(4-(1,3-dioxoisoindolin-2-yl)butyl) ethanethioate (2.38 g, 8.6 mmol) in ethanol (70 mL), and the resulting mixture was refluxed for 12 hours. After cooling to room temperature, the solvent was removed under reduced pressure, and the aqueous solution was extracted with CH_2Cl_2. After drying with Na_2SO_4, the organic layer was concentrated under reduced pressure. The light yellow oil product was obtained and used directly without further purification (360 mg, 40%). ESI MS (m/z): calcd. for $C_4H_{11}NS$, 105.1; found $[M+H]^+$, 106.1.

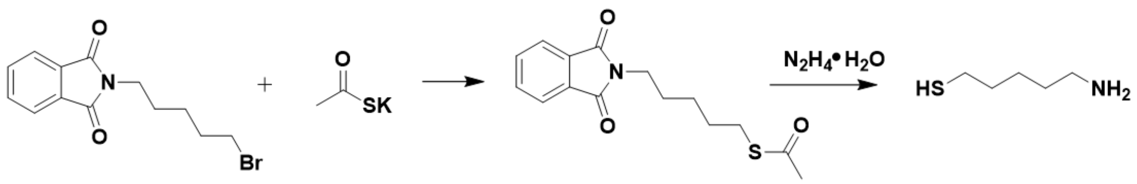

Synthesis of 5-aminopentane-1-thiol: 2-(5-bromopentyl)isoindoline-1,3-dione (2.55 g, 8.6 mmol) was suspended in THF (30 mL) and then added potassium thioacetate (0.98 g, 8.6 mmol). The mixture was refluxed 24 hours. After cooling to room temperature, the mixture was added into H_2O (30 mL), and the aqueous solution was extracted with EtOAc. The combined organic phases were dried with Na_2SO_4. The solvent was removed under reduced pressure to give the solid product (2.48 g, 99%), which was used directly without further purification. Hydrazine hydrate (2.6 mL, 67.6 mmol) was added into a solution of S-(5-(1,3-dioxoisoindolin-2-yl)pentyl) ethanethioate (2.48 g, 8.6 mmol) in ethanol (70 mL), and the resulting mixture was refluxed for 12 hours. After cooling to room temperature, the solvent was removed under reduced pressure, and the aqueous solution was extracted with CH_2Cl_2. After drying with Na_2SO_4, the organic layer was concentrated under reduced pressure. The light yellow oil product was obtained and used directly without further purification (285 mg, 28%). ESI MS (m/z): calcd. for $C_5H_{13}NS$, 119.1; found $[M+H]^+$, 120.1.

Synthesis of AuNPs, citrate-stabilized AuNPs, and different ligands-modified AuNPs. All glass containers are cleaned using the aqua regia solution. **Cautions:** aqua regia solution is very dangerous, should be operated after careful training and wearing protective equipment. Citrate-stabilized AuNPs with an approximate size of 13 nm were synthesized based on the reported protocol.[2] 100 mL deionized water containing $HAuCl_4$ (0.25 mM) was added into a 250 mL three-neck flask equipped with a water condenser. After boiling, 3.5 mL 1% sodium citrate solution was rapidly added under vigorous stirring and reflux conditions for 20 min. The cooled wine-red solution was centrifuged (12000 rpm, 20 min) to remove residual reagents and stored under 4 ^{o}C.

AuNPs with an approximate size of 22 nm were synthesized using AA as the reducing agent and stabilizing agent. 50 mL deionized water containing $HAuCl_4$ (0.25 mM) was added into a 100 mL round-bottom flask. After heated to 80 ^{o}C, 1 mL 100 mM

AA solution was rapidly added under vigorous stirring for 15 min. The cooled deep-red solution was immediately centrifuged (8000 rpm, 10 min) to remove residual reagents and stored under 4 ºC.

Different ligands-modified AuNPs were prepared by using a ligand exchange strategy. All ligand stock solutions are prepared by dissolving these molecules with a concentration of 10 mM and stored under -20 ºC. Typically, the properly diluted ligand solutions were mixed with AuNPs and placed under 4 ºC overnight (> 12 h).

Molecular modeling. The theoretical calculations of molecular length and distance of different ligands are performed using ChemBioOffice 2010 software (ChemBio3D Ultra 12.0) after MMFF94 minimization.

Calculation of surface coverage of AuNPs. According to the literature,[3-4] we calculated the total number of gold atoms in AuNPs based on complete volume filling. For spherical metal nanoparticles, the total number of gold atoms (N_T) in a nanoparticle can be calculated using the following equation:

$R = r_s N_T^{1/3}$,

R is the particle radius. r_s is the Wigner-Seitz radius, 0.145 nm for Au.

We took the TEM-measured size value as the representative data for the calculation.

For the 21.8 nm AuNPs (R = 10.9 nm), $N_T = (R / r_s)^3 = (10.9 / 0.145)^3 = 4.25 \times 10^5$.

The number of surface gold atoms per nanoparticle can be calculated using two different methods.

Method I: We calculated the number of surface gold atoms per nanoparticle (N_S) by an approximate estimation using the following equation:

$N_S \approx S_{NP} / S_{Au}$,

S_{NP} is the superficial area of nanoparticles. For spherical nanoparticles, $S_{NP} = \pi d^2$, d is the diameter of nanoparticle.

S_{Au} is the plane projected area of gold atom, $S_{Au} = \pi r_{Au}^2$, r_{Au} is the atomic radius of gold, 0.145 nm for Au.

For the 21.8 nm AuNPs (d = 21.8 nm), $N_S \approx (d / r_{Au})^2 = (21.8 / 0.145)^2 = 2.26 \times 10^4$.

Method II: The number of surface atoms per nanoparticle (N_s) depends on how many layers of gold atoms the particle is composed of.[5] It can be estimated using the following equation:

$N_s = 10n^2 + 2$,

n is the layer number. This is based on the close packing of the gold atoms in the nanoparticle. The smallest cluster (n = 1) has 13 atoms, 12 of which are surface atoms. Each additional layer adds ~ 0.238 nm to the nanoparticle radius. It can be estimated using the following equation:

$0.238(n + 0.5) \approx d / 2$,

d is the diameter of nanoparticle.

For the 21.8 nm AuNPs (d = 21.8 nm), $n \approx d / (0.238 \times 2) - 0.5 \approx 46$. $N_s = 10 \times 46^2 + 2 = 2.12 \times 10^4$.

Based on the above calculations, the average value of N_s is $(2.26 + 2.12) \times 10^4 / 2 = 2.19 \times 10^4$.

The surface gold atom ratio (n) is calculated using the following equation:

$n = (N_S / N_T)100\ \%$,

N_S is the number of surface gold atoms. N_T is the total number of gold atoms.

For **Method I**, $n_I = (2.26 \times 10^4 / 4.25 \times 10^5)\ 100\ \% = 5.32\ \%$.

For **Method II**, $n_{II} = (2.12 \times 10^4 / 4.25 \times 10^5)\ 100\ \% = 4.99\ \%$.

According to the above calculations based on two methods, we take the average value of the two methods as the estimated surface gold atom ratio. $n = (n_I + n_{II}) / 2 = 5.16\ \%$.

We calculated the nanoparticle concentration (C_{NP}) by assuming that over 50 % of gold ions are reduced into AuNPs. It can be estimated using the following equation:

$C_{NP} \approx 0.5C_{Au} / N_T$,

C_{Au} is the concentration of gold ions. N_T is the total gold atom number in a nanoparticle.

For the 21.8 nm AuNPs ($C_{Au} = 2.5 \times 10^{-4}$ M^{-1}, $N_T = 4.25 \times 10^5$), $C_{NP} \approx 0.5 \times 2.5 \times 10^{-4} / 4.25 \times 10^5 = 0.29 \times 10^{-9}$ M^{-1}. The concentration of AuNPs is about 0.29 nM.

We also calculated the nanoparticle concentration (C_{NP}) by using an approximate molar extinction coefficient. The molar extinction coefficient of 9.21×10^8 $M^{-1}cm^{-1}$ for 20 nm AuNPs (stabilized in citrate buffer) is referred to in the technical documents of Sigma-Aldrich. By assuming the same molar extinction coefficient for the 20 nm AuNPs and the 21.8 nm AuNPs, the calculated C_{NP} for the 21.8 nm AuNPs is about 0.44×10^{-9} M^{-1} (0.44 nM).

Based on the Au-S bond with Au : S ratio of 1 : 1 and the assuming of every surface gold atom forming Au-S bond, the minimum value of thiol ligand (C_{min}) to achieve 100 % surface coverage of AuNPs can be estimated using the following equation:

$C_{min} \approx C_{NP} N_S$,

C_{NP} is the nanoparticle concentration. N_S is the number of surface gold atoms.

For the 21.8 nm AuNPs ($C_{NP} \approx 0.29 \times 10^{-9}$ M^{-1}, $N_S \approx 2.19 \times 10^4$), $C_{min} \approx 0.29 \times 10^{-9} \times 2.19 \times 10^4 = 6.35 \times 10^{-6}$ M^{-1}. The required minimum concentration of thiol ligand is about 6.35 μM to achieve 100 % surface coverage of AuNPs.

In this work, 10 μM thiol ligand is used and satisfies the required minimum concentration to achieve 100 % surface coverage. Under this condition, the surface ligand density (ρ) is equal to the surface gold atom density. It can be estimated using the following equation:

$\rho = N_S / S_{NP}$,

N_S is the number of surface gold atoms. S_{NP} is the superficial area of nanoparticles.

For the 21.8 nm AuNPs ($N_S \approx 2.19 \times 10^4$, $S_{NP} = \pi d^2$), $\rho = 2.19 \times 10^4 / (3.14 \times 21.8^2) = 14.68\ nm^{-2}$. It is about 15 ligands per nm^{-2} on the surface of AuNPs. For the amine thiol ligands, the N elemental density (ρ_N) is equal to the ligand density. For amine thiol ligands, the N elemental density is about 15 per nm^{-2} on the surface of AuNPs.

Calculation based on the XPS tests. Taking **2N**-AuNPs and **11N**-AuNPs as the study models, the S : Au ratios of **2N**-AuNPs and **11N**-AuNPs are 10.43 % and 9.94 %, respectively. Under the 100 % surface coverage condition, the ligand number on the surface of AuNPs is equal to the surface gold atom number. Thus, the theoretical maximum S : Au ratio should ≤ n (the surface gold atom ratio). Based on the above calculations, for this work using 21.8 nm AuNPs, the n value is about 5.16 %. The XPS tested S :Au ratios of **2N**-AuNPs and **11N**-AuNPs are about 2-times higher than the theoretical value. On one hand, these high ratio values indicate that the ligands achieve the 100 % surface coverage of AuNPs. On the other hand, these overhigh values consist of the false signal owing to the residual ligands in the test sample (which are not anchored on the surface of AuNPs).

To evaluate the surface ligand of polyamine-AuNPs, we tested the N : Au ratio using XPS. The ligand density is simplified to N elemental density. For PEI-AuNPs, the XPS tested N : Au ratio is 27.25 %. The N elemental density (ρ_N) can be estimated using the following equation:

$\rho_N = (n_{N:Au} / n)\rho$,

$n_{N:Au}$ is the XPS tested N : Au ratio. n is the surface gold atom ratio. ρ is the surface gold atom density.

For the PEI-AuNPs ($n_{N:Au} = 27.25\ \%$, $n = 5.16\ \%$, $\rho = 14.68\ nm^{-2}$), $\rho_N = (27.25\ \% / 5.16\ \%) \times 14.68\ nm^{-2} = 77.53\ nm^{-2}$. It is about 78 N elements per nm^{-2} on the surface of PEI-AuNPs.

Table S1 N elemental density of different polyamine ligands on surfaces of AuNPs.

	PEI	PVAM	PAAM	PLL	ε-PLL	PAMAM
N : Au (%)	27.25	26.33	25.05	22.44	22.96	20.16
N density (nm^{-2})	78	75	71	64	65	57

POX-mimic activity tests. For the tests of POX-mimic activity, 100 μL commercial substrate solution of TMB/H_2O_2 was mixed with 100 μL AuNPs and different ligands-modified AuNPs. The mixtures were incubated at ambient conditions. OD_{650nm} was monitored, and the endpoint optical spectra were recorded.

Hydrogen peroxide and glucose tests. TMB was dissolved in DMF with a concentration of 50 mM as the stock solution and stored under 4 ºC. Glucose was dissolved in deionized water with a concentration of 100 mM as the stock solution and stored under 4 ºC. GOx was dissolved in deionized water with a concentration of 6 KU/mL as the stock solution and stored under -20 ºC. Tris-HCl buffer (pH 7.0, 10 mM) and acetate buffer (pH 4.0, 200 mM) solutions were used as reaction buffer referenced by gold protocols and reported works.[6-7] For the tests of H_2O_2, 100 μL H_2O_2 solution with different concentrations were mixed with 5 μL TMB solution and 100 μL acetate buffer solution. Then 50 μL AuNPs or PEI-AuNPs was added into the mixtures and incubated under dark conditions. For the tests of glucose, glucose solutions with different concentrations were prepared by diluting with Tris-HCl buffer. 2.5 μL GOx solution was mixed with different glucose solutions and incubated at 37 ºC for 1 h. Then 5 μL TMB solution, 100 μL acetate buffer solution, and 50 μL PEI-AuNPs were added into the mixtures and incubated at 37 ºC for 2 h under dark conditions. After incubation, the optical spectra were recorded.

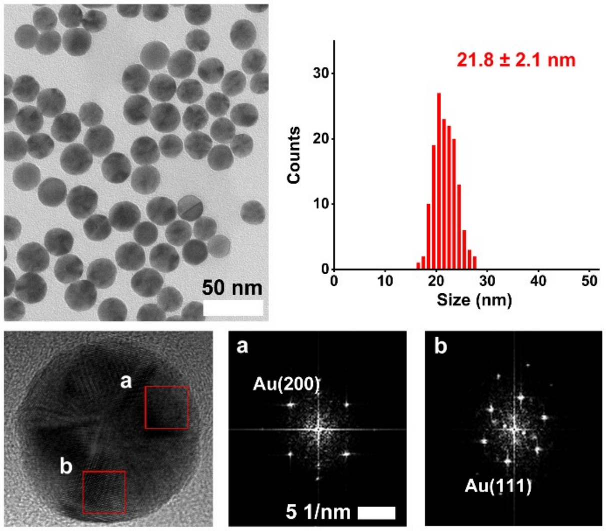

Fig. S1 TEM, size distribution profile, HRTEM, and selected area fast Fourier transform (FFT) pattern images of AuNPs.

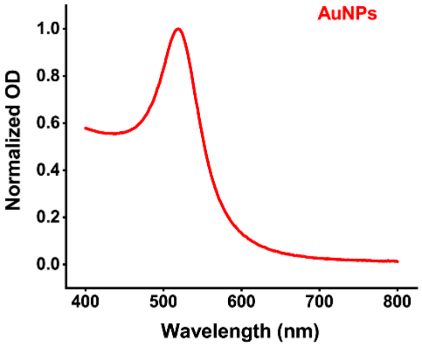

Fig. S2 The normalized optical spectrum of AuNPs.

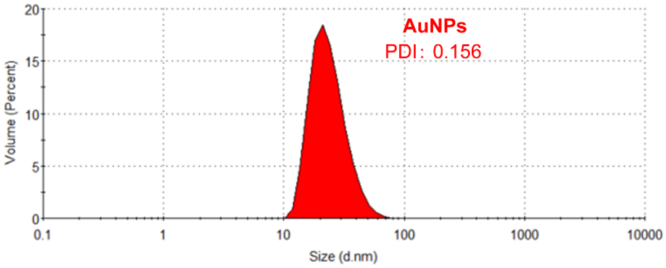

Fig. S3 Dynamic light scattering (DLS) size profile of AuNPs (24.1 ± 8.4 nm).

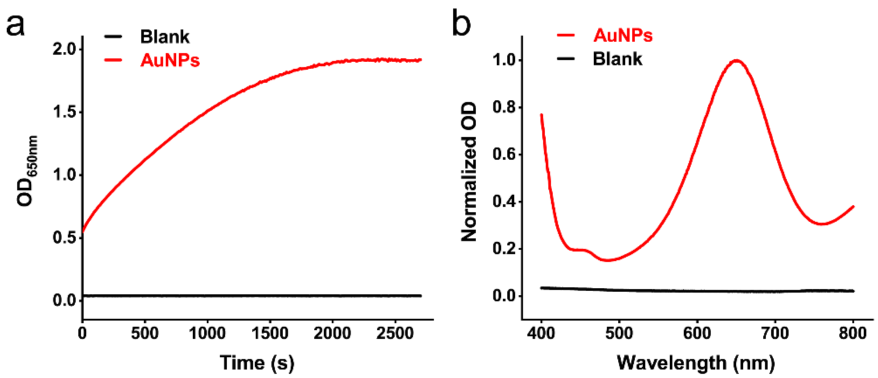

Fig. S4 POX-mimic activity tests. (a) Kinetic monitoring of AuNPs in the presence of substrate TMB/H_2O_2 at 650nm. (b) The normalized optical spectrum of AuNPs after incubation with substrate TMB/H_2O_2.

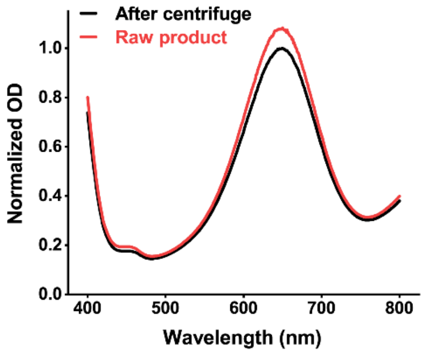

Fig. S5 The normalized optical spectra of raw AuNPs and centrifuged AuNPs after incubation with substrate TMB/H_2O_2.

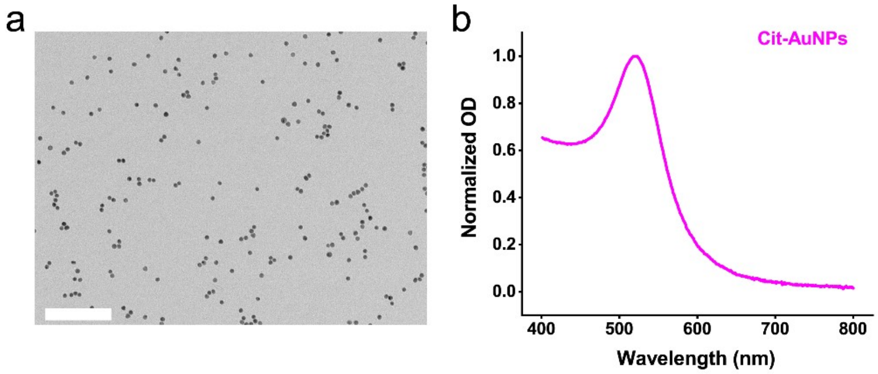

Fig. S6 Characterizations of citrate-stabilized AuNPs (Cit-AuNPs). (a) TEM image of the Cit-AuNPs. Scale bar: 200 nm. (b) The normalized optical spectrum of Cit-AuNPs.

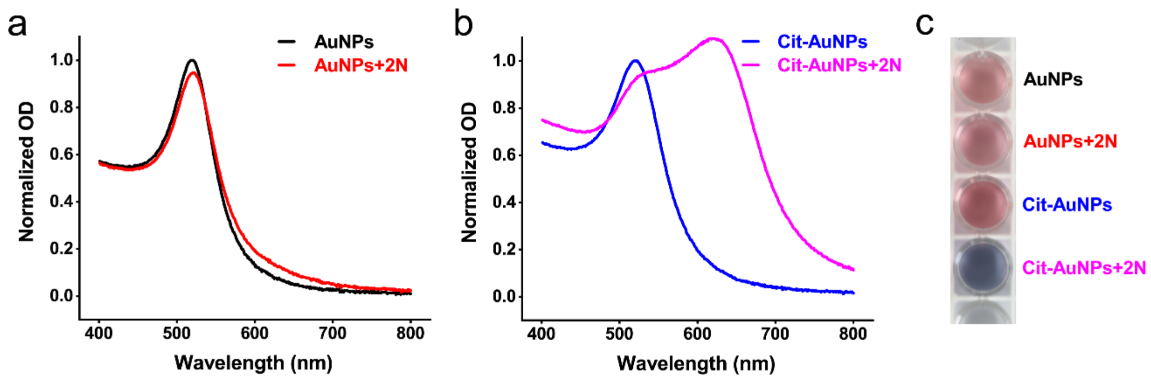

Fig. S7 Optical responses of AuNPs and citrate-stabilized AuNPs (Cit-AuNPs). (a) and (b) Normalized optical spectra of AuNPs and Cit-AuNPs in the absence and presence of **2N** (100 μM). (c) The corresponding photograph.

Table S2 Physicochemical information of different amine ligands.

Ligand	Solvent accessible Ball&Stick mode	Ionized group	Molecular length/ Distance (Å)	Symbol
2-aminoethanethiol (C_2H_7NS)	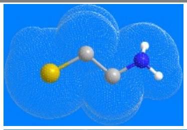	$-NH_3^+$ (positive)	5.7 / 4.1	**2N**
3-aminopropane-1-thiol (C_3H_9NS)	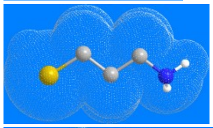	$-NH_3^+$ (positive)	6.9 / 5.2	**3N**
4-aminobutane-1-thiol ($C_4H_{11}NS$)	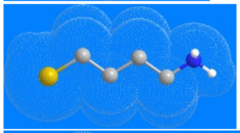	$-NH_3^+$ (positive)	8.2 / 6.5	**4N**
5-aminopentane-1-thiol ($C_5H_{13}NS$)	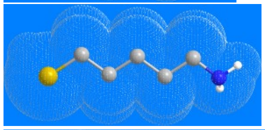	$-NH_3^+$ (positive)	9.4 / 7.7	**5N**
6-aminohexane-1-thiol ($C_6H_{15}NS$)	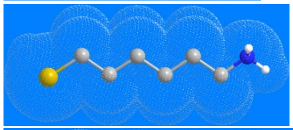	$-NH_3^+$ (positive)	10.7 / 9.0	**6N**
8-aminooctane-1-thiol ($C_8H_{19}NS$)	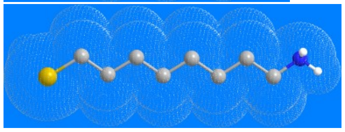	$-NH_3^+$ (positive)	13.2 / 11.6	**8N**
11-aminoundecane-1-thiol ($C_{11}H_{25}NS$)	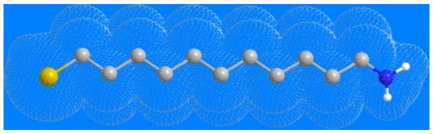	$-NH_3^+$ (positive)	17.0 / 15.3	**11N**

Table S3 Physicochemical information of different ethanethiol ligands.

Ligand	Solvent accessible Ball&Stick mode	Ionized group	Molecular length/ Distance (Å)	Symbol
2-mercapto-N,N,N-trimethylethanaminium ($C_5H_{14}NS$)	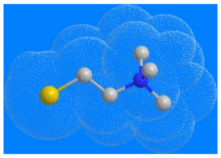	$-N(CH_3)_3^+$ (positive)	7.1 / 4.2	**$2N^+$**
2-mercaptoethanol (C_2H_6OS)	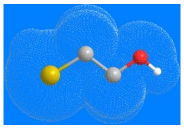	/	5.6 / 4.0	**2OH**
3-mercaptopropanoic acid ($C_3H_6O_2S$)	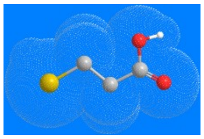	$-COO^-$ (negative)	6.1 / 4.1	**2COOH**
2-mercaptoethanesulfonic acid ($C_2H_6O_3S_2$)	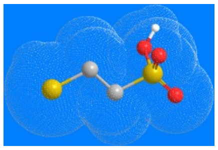	$-SO_3^-$ (negative)	6.3 / 4.4	**$2SO_3H$**

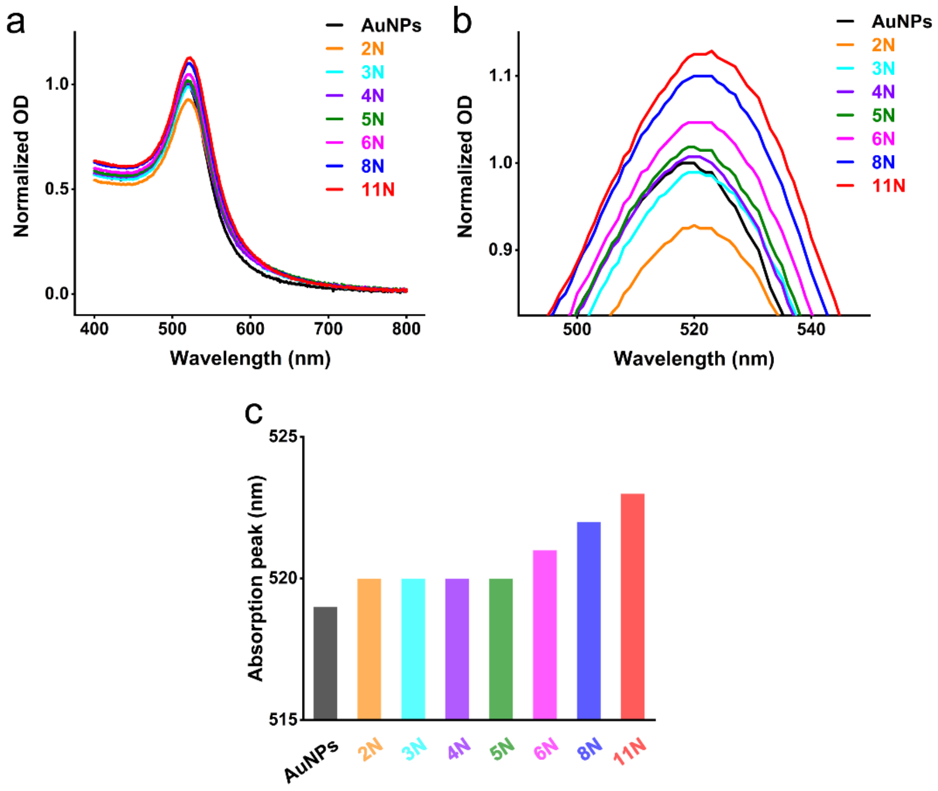

Fig. S8 Characterizations of different ligands modified-AuNPs. (a) Normalized optical spectra of different amine ligands modified-AuNPs. (b) The corresponding enlarged region. (c) The corresponding redshift of the absorption peak (a redshift of 4 nm for **11N-**AuNPs).

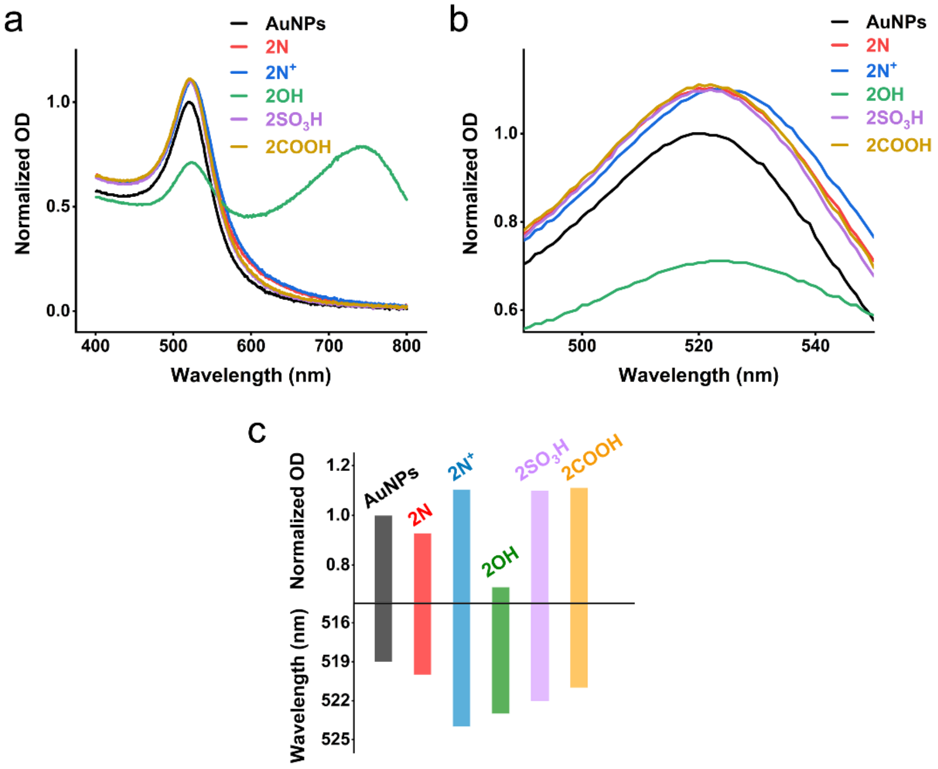

Fig. S9 Characterizations of different ligands modified-AuNPs. (a) Normalized optical spectra of different ethanethiol ligands modified-AuNPs. (b) The corresponding enlarged region. (c) The corresponding absorption peak value and the normalized optical absorption data.

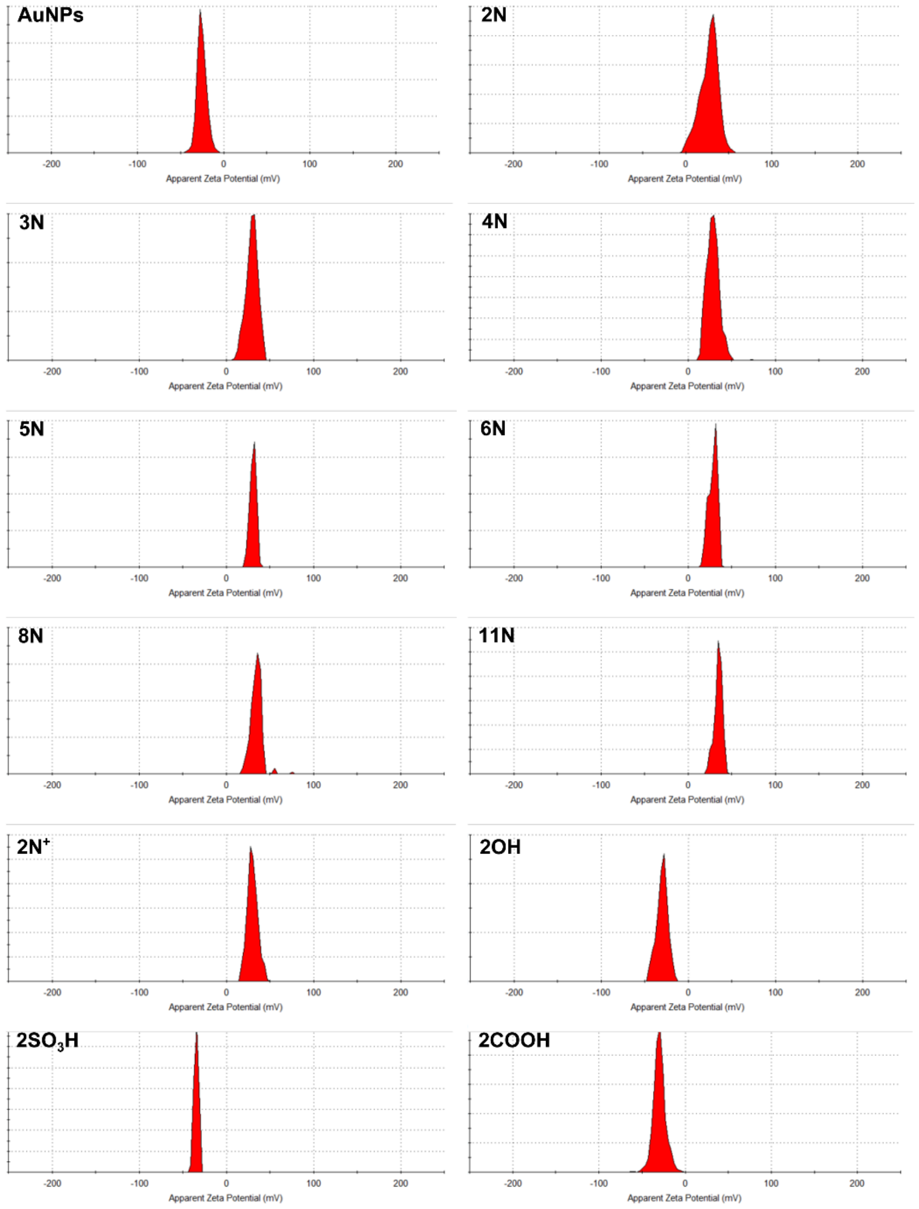

Fig. S10 Zeta-potential distribution profiles of AuNPs and different ligands modified-AuNPs.

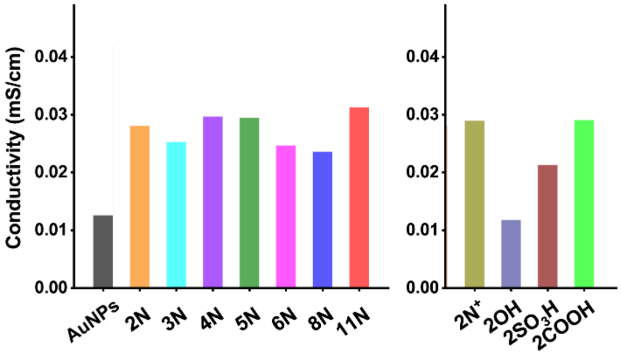

Fig. S11 Conductivity data of AuNPs and different ligands modified-AuNPs solution.

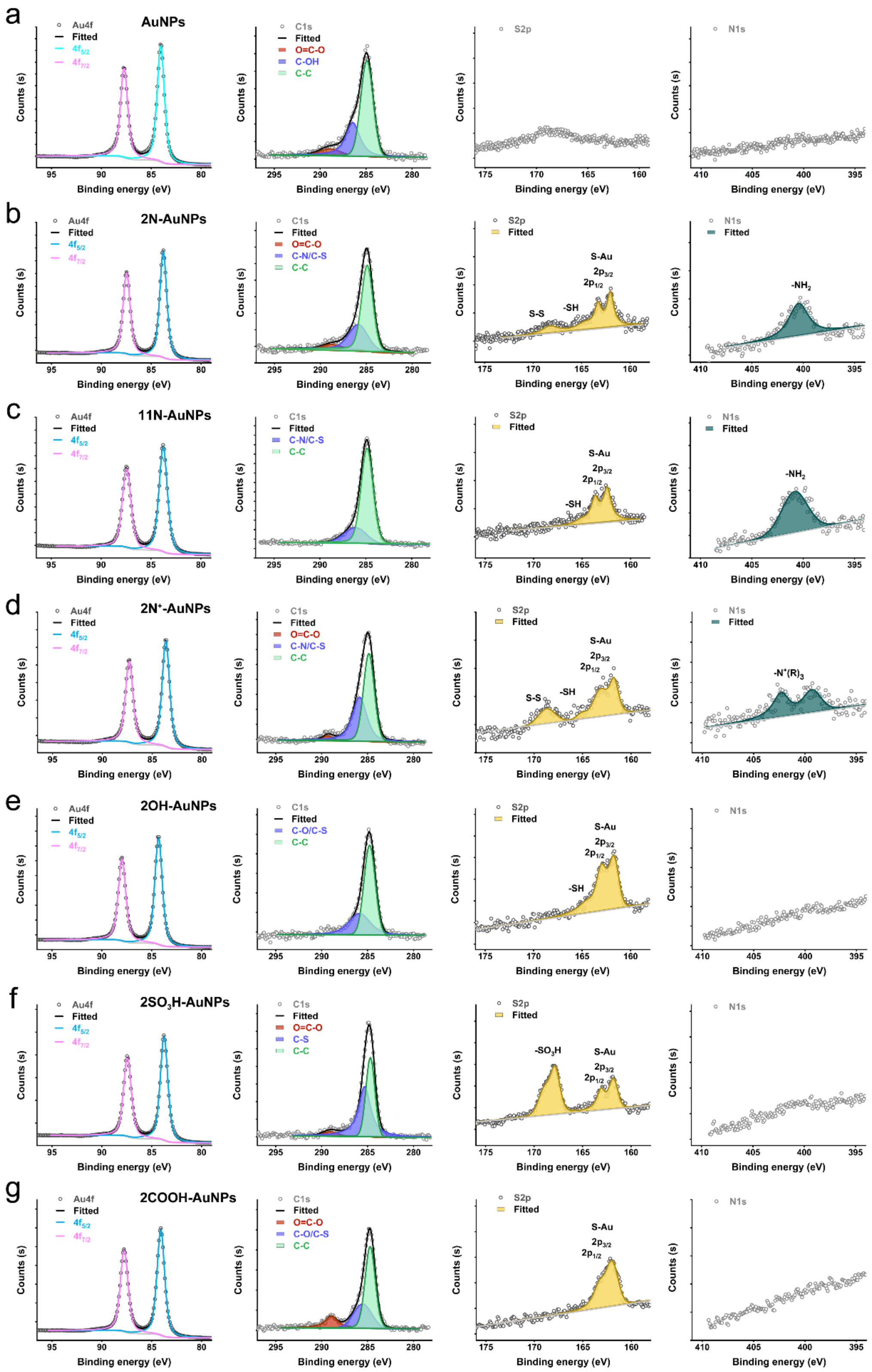

Fig. S12 Characterizations of different ligands modified-AuNPs. XPS elemental binding energy profiles of AuNPs and different ligands modified-AuNPs.

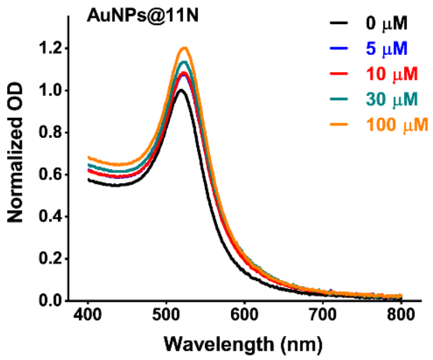

Fig. S13. Normalized optical spectra of different concentrations of **11N**-incubated AuNPs.

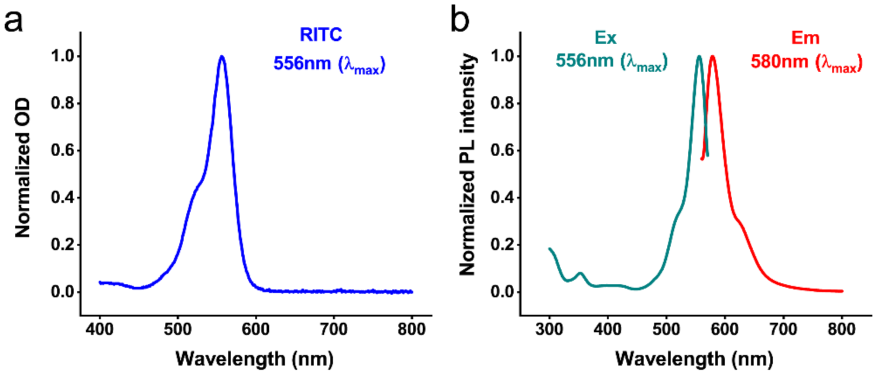

Fig. S14 Characterizations of RITC. (a) The normalized optical spectrum of RITC. (b) The normalized excitation (Ex) and emission (Em) spectra of RITC.

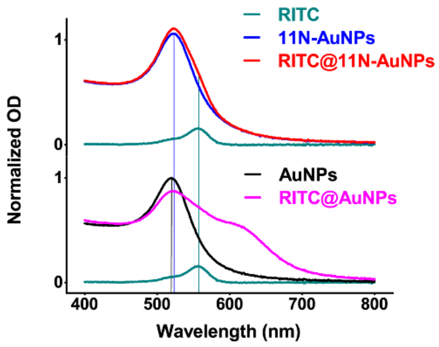

Fig. S15 The normalized optical spectra of AuNPs and **11N**-AuNPs before and after incubation with RITC.

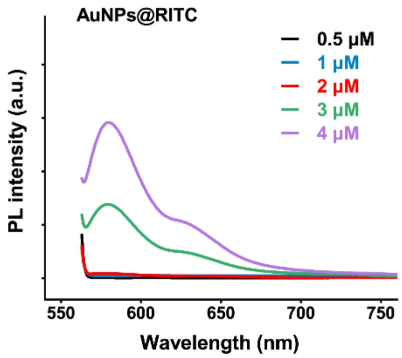

Fig. S16 Fluorescence spectra of different amounts of RITC when incubation with AuNPs.

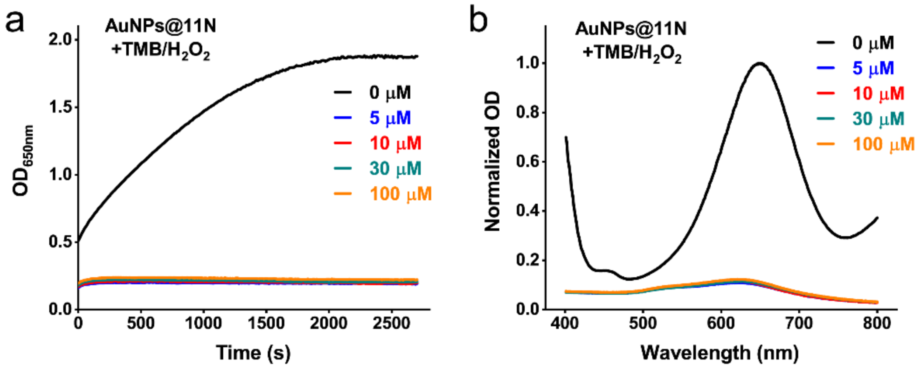

Fig. S17 Characterizations of **11N**-AuNPs. (a) Kinetic monitoring of different concentrations of **11N**-incubated AuNPs in the presence of substrate TMB/H_2O_2. (b) Normalized optical spectra of different concentrations of **11N**-incubated AuNPs after incubation with substrate TMB/H_2O_2.

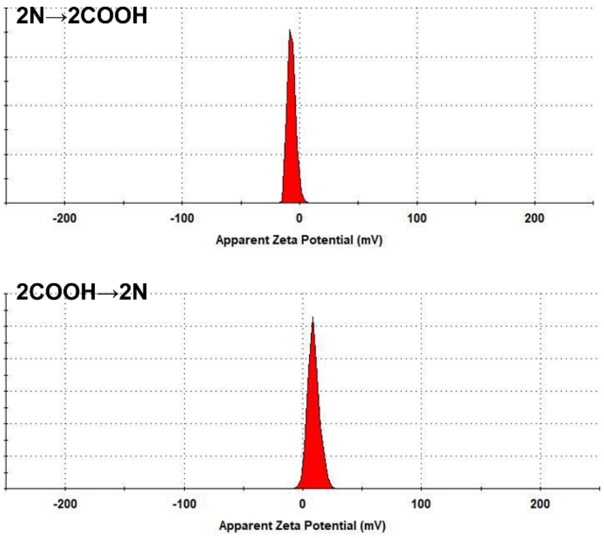

Fig. S18 Zeta-potential distribution profiles of **2N**-AuNPs and **2COOH**-AuNPs after incubation with **2COOH** and **2N** (200 µM, 1 hour), respectively.

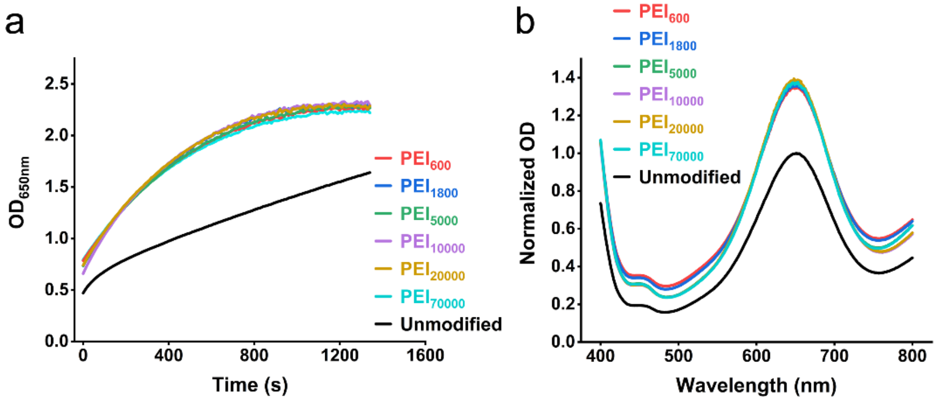

Fig. S19 Characterizations of PEI-AuNPs. (a) Kinetic monitoring of different PEI-modified AuNPs in the presence of substrate TMB/H_2O_2. (b) Normalized optical spectra of different PEI-AuNPs after incubation with substrate TMB/H_2O_2.

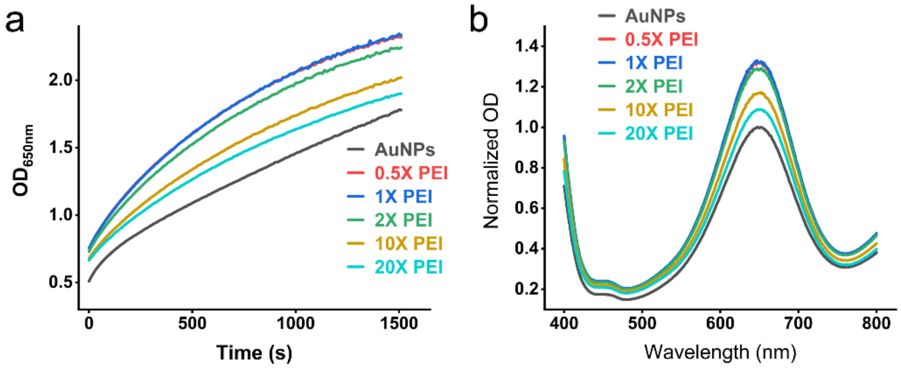

Fig. S20 Characterizations of PEI-AuNPs. (a) Kinetic monitoring of different concentrations of PEI-modified AuNPs in the presence of substrate TMB/H_2O_2. (b) Normalized optical spectra of different concentrations of PEI-modified AuNPs after incubation with substrate TMB/H_2O_2. 1X = 3 μg/mL.

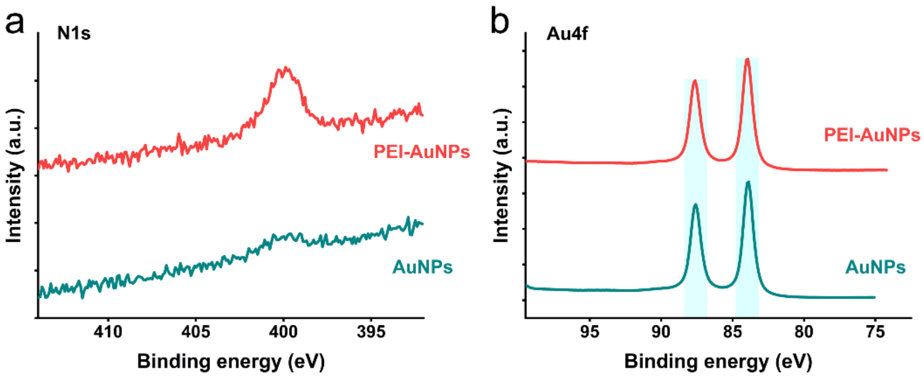

Fig. S21 XPS characterizations of PEI-AuNPs. (a) and (b) Au4f and N1s binding energy profiles of AuNPs and PEI-AuNPs, respectively.

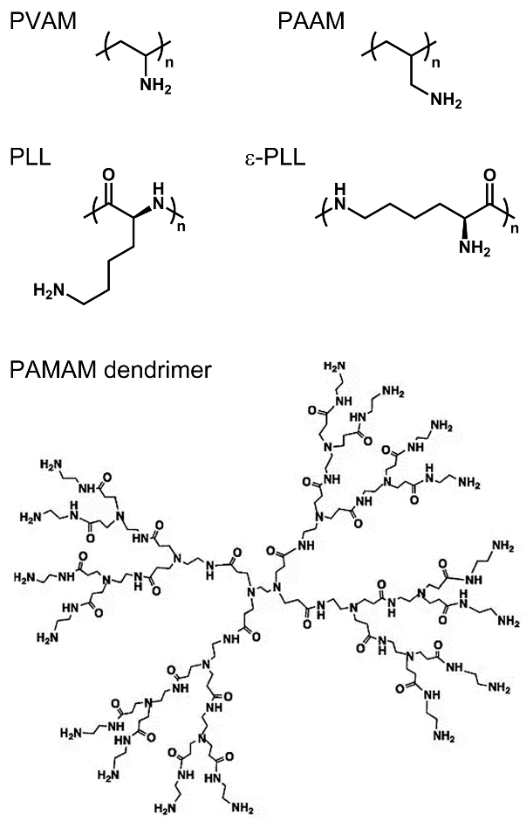

Fig. S22 Chemical structure of different polyamine ligands.

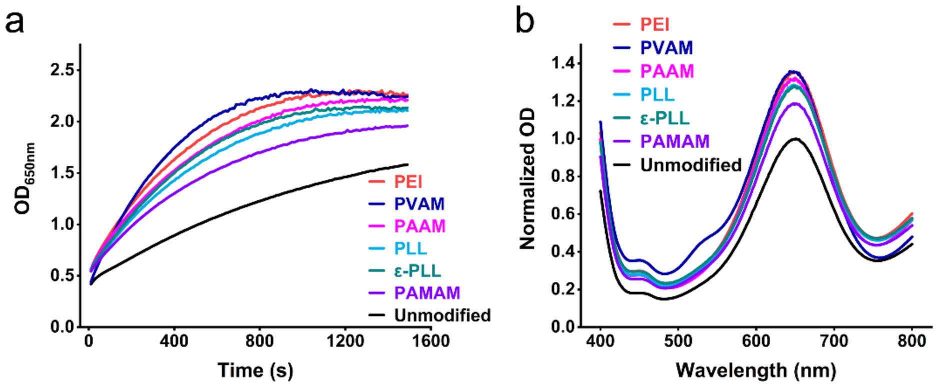

Fig. S23 Characterizations of polymer-modified AuNPs. (a) Kinetic monitoring of different polymer-modified AuNPs in the presence of substrate TMB/H_2O_2. (b) Normalized optical spectra of different polymer-modified AuNPs after incubation with substrate TMB/H_2O_2.

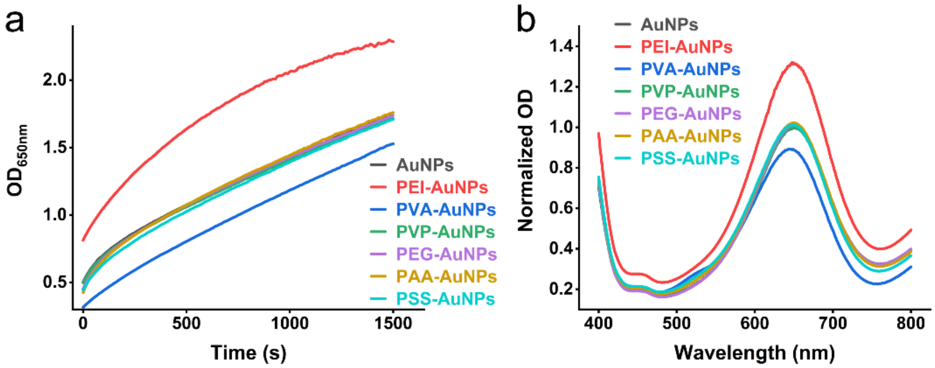

Fig. S24 Characterizations of polymer-modified AuNPs. (a) Kinetic monitoring of different polymer-modified AuNPs in the presence of substrate TMB/H_2O_2. (b) Normalized optical spectra of different polymer-modified AuNPs after incubation with substrate TMB/H_2O_2.

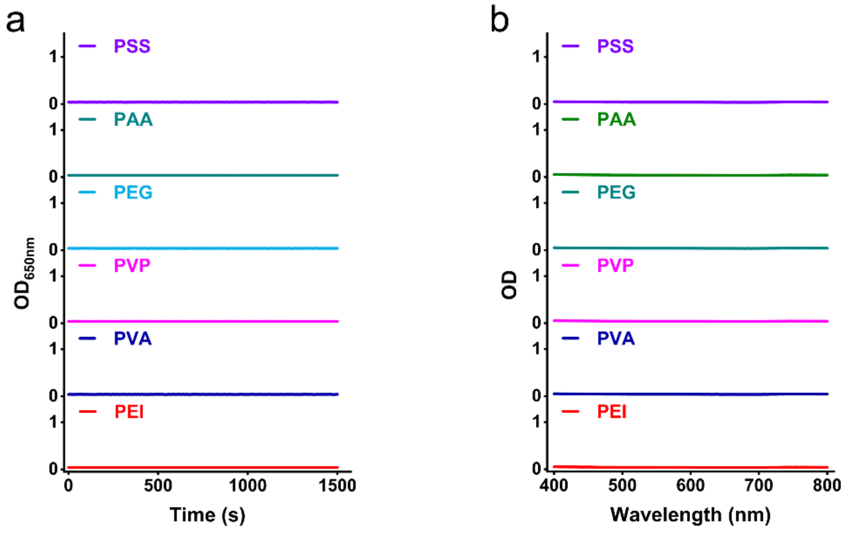

Fig. S25 POX activity tests of different polymers. (a) and (b) Kinetic monitoring and endpoint optical spectra of different polymers in the presence of substrate TMB/H_2O_2, respectively.

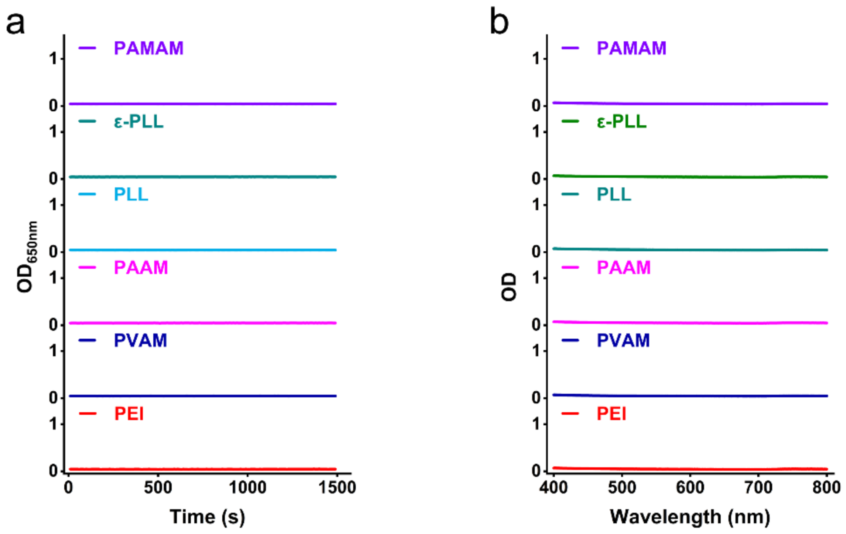

Fig. S26 POX activity tests of different polymers. (a) and (b) Kinetic monitoring and endpoint optical spectra of different polymers in the presence of substrate TMB/H_2O_2, respectively.

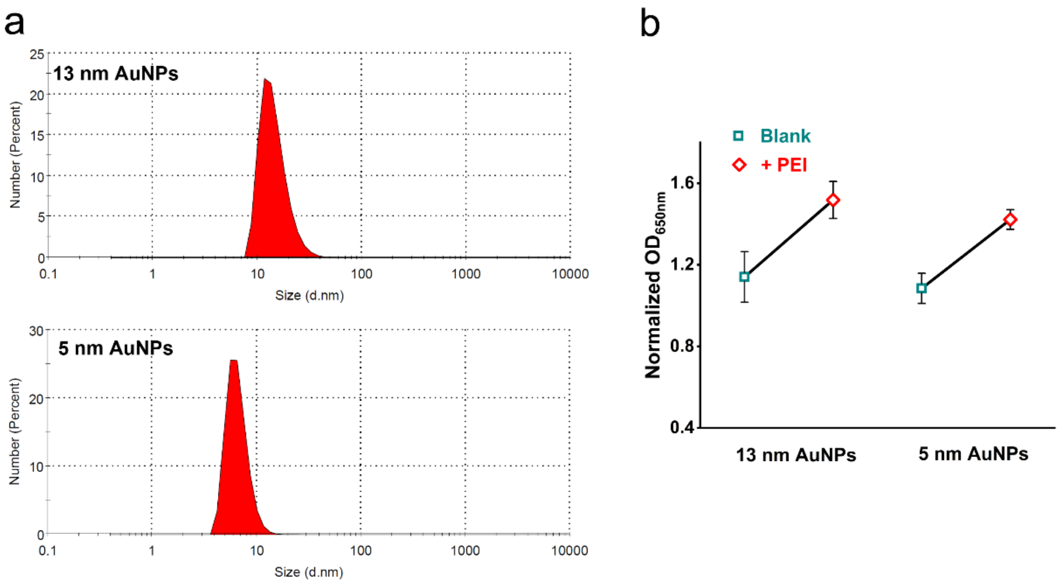

Fig. S27 POX activity tests of different size AuNPs. (a) Dynamic light scattering diameter profiles of the used ~ 13 nm AuNPs and ~ 5 nm AuNPs. (b) The normalized absorption at 650 nm of different size AuNPs in the absence and presence of PEI after incubation with substrate TMB/H_2O_2.

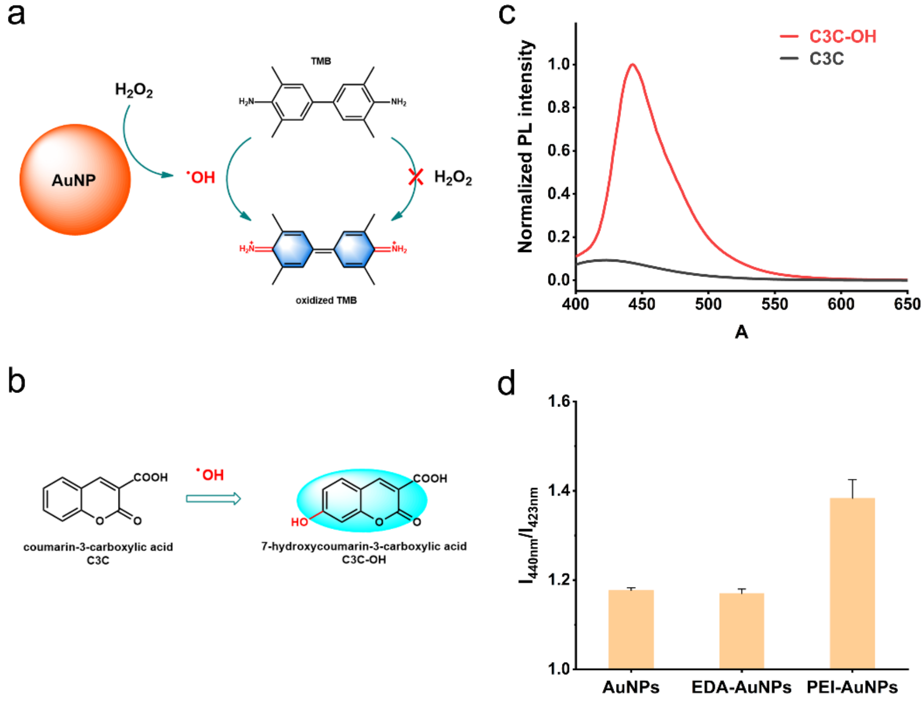

Fig. S28 Oxygen radicals tests. (a) Schematic illustration of AuNP catalyzing H_2O_2 to generate hydroxyl radical (˙OH) to facilitate the oxidation of TMB. (b) Schematic illustration of the oxidation of coumarin-3-carboxylic acid (C3C) to produce fluorescent 7-hydroxycoumarin -3-carboxylic acid (C3C-OH) by ˙OH. (c) The normalized fluorescence spectra of C3C and C3C-OH (excitation at 356 nm). (d) The fluorescence intensity ratio of C3C after incubation with AuNPs, EDA-AuNPs, and PEI-AuNPs in the presence of H_2O_2.

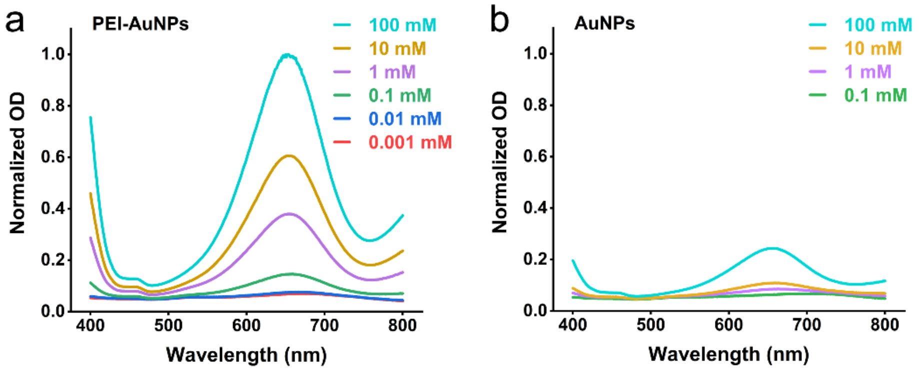

Fig. S29 H_2O_2 tests using PEI-modified AuNPs and AuNPs. (a) Normalized optical spectra of PEI-modified AuNPs respond to different concentrations of H_2O_2 in the presence of TMB. (b) Normalized optical spectra of AuNPs respond to different concentrations of H_2O_2 in the presence of TMB.

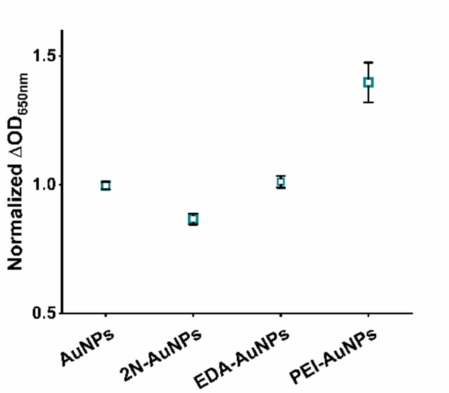

Fig. S30 Comparision studies of AuNPs, **2N**-AuNPs, EDA-AuNPs, and PEI-AuNPs responding to the same amount of H_2O_2 to oxidize TMB substrate.

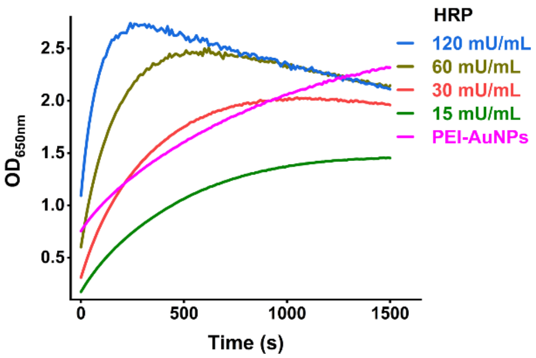

Fig. S31 POX activity comparison between HRP and PEI-AuNPs. Kinetic monitoring of different amounts of HRP and PEI-AuNPs in the presence of substrate TMB/H_2O_2.

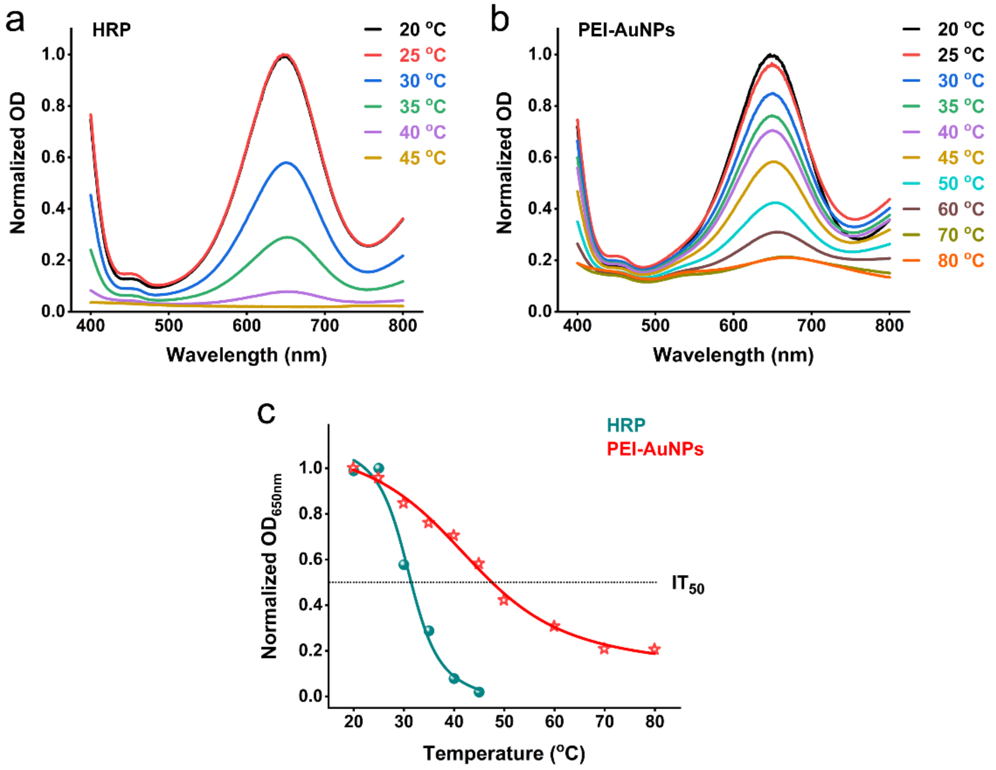

Fig. S32 Temperature-dependent POX activity of HRP and PEI-AuNPs. (a) and (b) Normalized optical spectra of 100 ng/mL HRP and PEI-AuNPs after incubation with substrate TMB/H_2O_2 at different temperatures, respectively. (c) The corresponding temperature-dependent curves of normalized absorption at 650 nm.

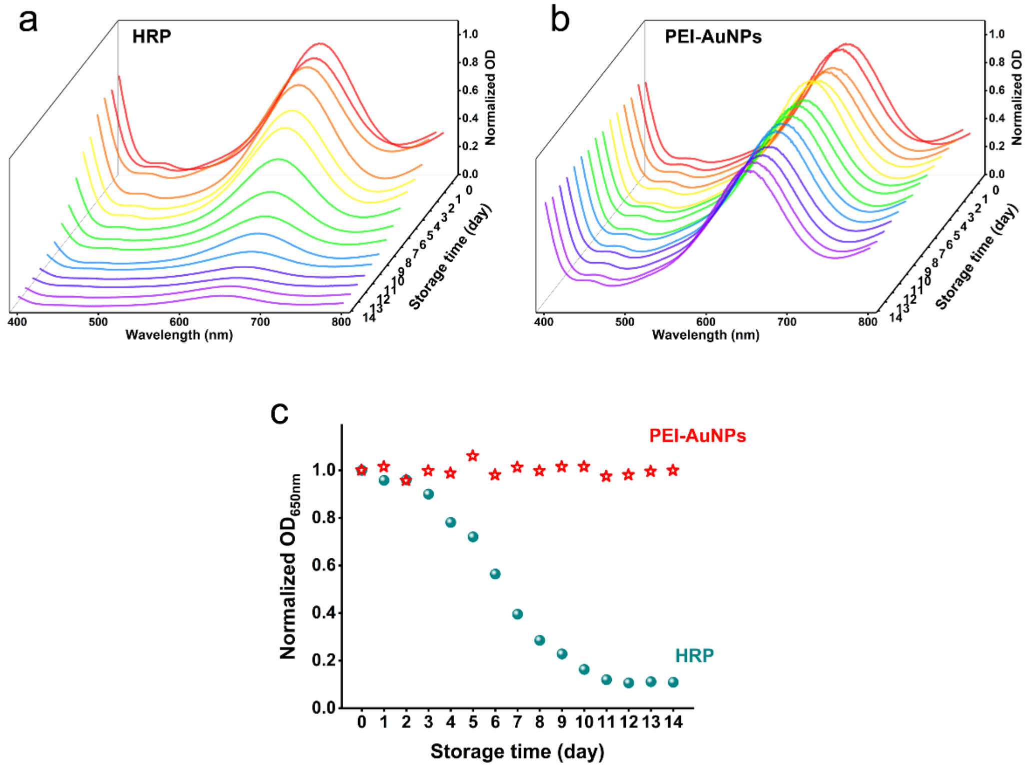

Fig. S33 Time-dependent POX activity of HRP and PEI-AuNPs. (a) and (b) Normalized optical spectra of 100 ng/mL HRP and PEI-AuNPs after incubation with substrate TMB/H_2O_2 at different storage times (room temperature), respectively. (c) The corresponding time-dependent curves of normalized absorption at 650 nm.

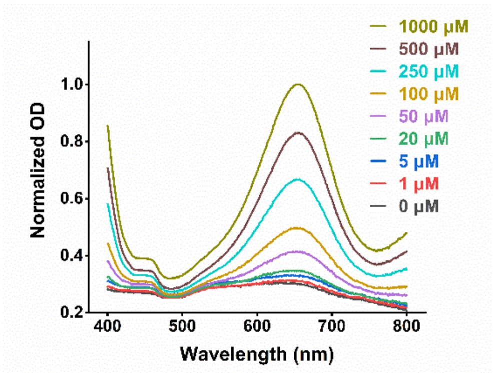

Fig. S34 Glucose tests using PEI-modified AuNPs. Normalized optical spectra of PEI-modified AuNPs respond to different concentrations of glucose in the presence of GOx and TMB.

Table S4 Analytical performance of different glucose sensors.

Materials / Methods	Limit of quantification (LOQ, mM)	Detection range (mM) / Orders of magnitude	Ref.
GO-COOH&GOx / Colorimetric	0.001	0.001 ~ 0.02 / 1	7
TTA-UCNP / Fluorometric	~ 0.3	0.3 ~ 16.7 / 2	8
Au&PB&GOx / Electrochemistry	0.005	0.005 ~ 0.1 / 2	9
$BPCNF_{900}$&GOx / Electrochemistry	0.01	0.01 ~ 3.8 / 2	10
ZIF-8(NiPd)&GOx / Electrochemistry	0.01	0.01 ~ 0.3 / 1	11
AuNPs&MIL-101&GOx / SERS	0.01	0.01 ~ 0.2 / 1	12
g-C_3N_4 / Colorimetric	10	10 ~ 300 / 1	13
BSA-MnO_2 NFs / Colorimetric	0.005	0.005 ~ 1.2 / 3	14
$Ni_{60}Nb_{40}$ Nanoglass / Electrochemistry	0.25	0.25 ~ 4 / 1	15
CeO_2&DNA&GOx / Fluorometric	0.01	0.01 ~ 0.2 / 1	16
AuFON&1,1-BBA / SERS	1	1 ~ 10 / 1	17
PBA-PAM hydrogel / Transmittance	5	5 ~ 50 / 1	18
PtNPs&GOx&PAni hydrogel / Colorimetric	0.01	0.01 ~ 8 / 2	19
GDY&Fe&GOx / Colorimetric	0.005	0.005 ~ 0.16 / 2	20
PEI-AuNPs&GOx / Colorimetric	0.00078	0.001 ~ 1 mM / 3	This work

References:

(1) Wang, X.; Zhao, X.; Dong, W.; Zhang, X.; Xiang, Y.; Huang, Q.; Chen, H., Integrating Amino Groups within Conjugated Microporous Polymers by Versatile Thiol–Yne Coupling for Light-Driven Hydrogen Evolution. *J. Mater. Chem. A* **2019,** *7* (27), 16277-16284.

(2) Gao, Y.; Zou, F.; Wu, B.; Wang, X.; Zhang, J.; Koh, K.; Chen, H., CB[7]-Mediated Signal Amplification Approach for Sensitive Surface Plasmon Resonance Spectroscopy. *Biosens. Bioelectron.* **2016,** *81*, 207-213.

(3) Gong, L.; Chen, Y.; He, K.; Liu, J., Surface Coverage-Regulated Cellular Interaction of Ultrasmall Luminescent Gold Nanoparticles. *ACS Nano* **2019,** *13* (2), 1893-1899.

(4) Xie, Y.; Yang, J.; Zhang, J.; Zheng, W.; Jiang, X., Activating the Antibacterial Effect of 4,6-Diamino-2-Pyrimidinethiol-Modified Gold Nanoparticles by Reducing their Sizes. *Angew. Chem. Int. Ed.* **2020,** *59* (52), 23471-23475.

(5) Mei, B. C.; Oh, E.; Susumu, K.; Farrell, D.; Mountziaris, T. J.; Mattoussi, H., Effects of Ligand Coordination Number and Surface Curvature on the Stability of Gold Nanoparticles in Aqueous Solutions. *Langmuir* **2009,** *25* (18), 10604-10611.

(6) Jv, Y.; Li, B.; Cao, R., Positively-Charged Gold Nanoparticles as Peroxidiase Mimic and Their Application in Hydrogen Peroxide and Glucose Detection. *Chem. Commun.* **2010,** *46* (42), 8017-8019.

(7) Song, Y.; Qu, K.; Zhao, C.; Ren, J.; Qu, X., Graphene Oxide: Intrinsic Peroxidase Catalytic Activity and Its Application to Glucose Detection. *Adv. Mater.* **2010,** *22* (19), 2206-2210.

(8) Huang, L.; Le, T.; Huang, K.; Han, G., Enzymatic Enhancing of Triplet–Triplet Annihilation Upconversion by Breaking Oxygen Quenching for Background-Free Biological Sensing. *Nat. Commun.* **2021,** *12* (1), 1898.

(9) Chen, Y.; Lu, S.; Zhang, S.; Li, Y.; Qu, Z.; Chen, Y.; Lu, B.; Wang, X.; Feng, X., Skin-Like Biosensor System via Electrochemical Channels for Noninvasive Blood Glucose Monitoring. *Sci. Adv.* **2017,** *3* (12), e1701629.

(10) Liang, T.; Zou, L.; Guo, X.; Ma, X.; Zhang, C.; Zou, Z.; Zhang, Y.; Hu, F.; Lu, Z.; Tang, K.; Li, C. M., Rising Mesopores to Realize Direct Electrochemistry of Glucose Oxidase toward Highly Sensitive Detection of Glucose. *Adv. Funct. Mater.* **2019,** *29* (44), 1903026.

(11) Wang, Q.; Zhang, X.; Huang, L.; Zhang, Z.; Dong, S., GOX@ZIF-8(NiPd) Nanoflower: An Artificial Enzyme System for Tandem Catalysis. *Angew. Chem. Int. Ed.* **2017,** *56* (50), 16082-16085.

(12) Hu, Y.; Cheng, H.; Zhao, X.; Wu, J.; Muhammad, F.; Lin, S.; He, J.; Zhou, L.; Zhang, C.; Deng, Y.; Wang, P.; Zhou, Z.; Nie, S.; Wei, H., Surface-Enhanced Raman Scattering Active Gold Nanoparticles with Enzyme-Mimicking Activities for Measuring Glucose and Lactate in Living Tissues. *ACS Nano* **2017,** *11* (6), 5558-5566.

(13) Zhang, P.; Sun, D.; Cho, A.; Weon, S.; Lee, S.; Lee, J.; Han, J. W.; Kim, D.-P.; Choi, W., Modified Carbon Nitride Nanozyme as Bifunctional Glucose Oxidase-Peroxidase for Metal-Free Bioinspired Cascade Photocatalysis. *Nat. Commun.* **2019,** *10* (1), 940.

(14) Han, L.; Zhang, H.; Chen, D.; Li, F., Protein-Directed Metal Oxide Nanoflakes with Tandem Enzyme-Like Characteristics: Colorimetric Glucose Sensing Based on One-Pot Enzyme-Free

Cascade Catalysis. *Adv. Funct. Mater.* **2018,** *28* (17), 1800018.
(15) Bag, S.; Baksi, A.; Nandam, S. H.; Wang, D.; Ye, X.; Ghosh, J.; Pradeep, T.; Hahn, H., Nonenzymatic Glucose Sensing Using $Ni_{60}Nb_{40}$ Nanoglass. *ACS Nano* **2020,** *14* (5), 5543-5552.
(16) Liu, B.; Sun, Z.; Huang, P.-J. J.; Liu, J., Hydrogen Peroxide Displacing DNA from Nanoceria: Mechanism and Detection of Glucose in Serum. *J. Am. Chem. Soc.* **2015,** *137* (3), 1290-1295.
(17) Sharma, B.; Bugga, P.; Madison, L. R.; Henry, A.-I.; Blaber, M. G.; Greeneltch, N. G.; Chiang, N.; Mrksich, M.; Schatz, G. C.; Van Duyne, R. P., Bisboronic Acids for Selective, Physiologically Relevant Direct Glucose Sensing with Surface-Enhanced Raman Spectroscopy. *J. Am. Chem. Soc.* **2016,** *138* (42), 13952-13959.
(18) Elsherif, M.; Hassan, M. U.; Yetisen, A. K.; Butt, H., Glucose Sensing with Phenylboronic Acid Functionalized Hydrogel-Based Optical Diffusers. *ACS Nano* **2018,** *12* (3), 2283-2291.
(19) Zhai, D.; Liu, B.; Shi, Y.; Pan, L.; Wang, Y.; Li, W.; Zhang, R.; Yu, G., Highly Sensitive Glucose Sensor Based on Pt Nanoparticle/Polyaniline Hydrogel Heterostructures. *ACS Nano* **2013,** *7* (4), 3540-3546.
(20) Liu, J.; Shen, X.; Baimanov, D.; Wang, L.; Xiao, Y.; Liu, H.; Li, Y.; Gao, X.; Zhao, Y.; Chen, C., Immobilized Ferrous Ion and Glucose Oxidase on Graphdiyne and its Application on One-Step Glucose Detection. *ACS Appl. Mater. Interfaces* **2019,** *11* (3), 2647-2654.
